# L-Norvaline Reverses Cognitive Decline and Synaptic Loss in a Murine Model of Alzheimer’s Disease

**DOI:** 10.1007/s13311-018-0669-5

**Published:** 2018-10-04

**Authors:** Baruh Polis, Kolluru D. Srikanth, Evan Elliott, Hava Gil-Henn, Abraham O. Samson

**Affiliations:** 1 Drug Discovery Laboratory, The Azrieli Faculty of Medicine, Bar-Ilan University, 1311502 Safed, Israel; 2 Laboratory of Cell Migration and Invasion, The Azrieli Faculty of Medicine, Bar-Ilan University, 1311502 Safed, Israel; 3 Laboratory of Molecular and Behavioral Neuroscience, The Azrieli Faculty of Medicine, Bar-Ilan University, 8th Henrietta Szold Street, P.O. Box 1589, 1311502 Safed, Israel

**Keywords:** Alzheimer’s disease, L-norvaline, L-arginine, arginase, ribosomal protein S6 kinase β-1, mTOR.

## Abstract

The urea cycle is strongly implicated in the pathogenesis of Alzheimer’s disease (AD). Arginase-I (ARGI) accumulation at sites of amyloid-beta (Aβ) deposition is associated with L-arginine deprivation and neurodegeneration. An interaction between the arginase II (ARGII) and mTOR-ribosomal protein S6 kinase β-1 (S6K1) pathways promotes inflammation and oxidative stress. In this study, we treated triple-transgenic (3×Tg) mice exhibiting increased S6K1 activity and wild-type (WT) mice with L-norvaline, which inhibits both arginase and S6K1. The acquisition of spatial memory was significantly improved in the treated 3×Tg mice, and the improvement was associated with a substantial reduction in microgliosis. In these mice, increases in the density of dendritic spines and expression levels of neuroplasticity-related proteins were followed by a decline in the levels of Aβ toxic oligomeric and fibrillar species in the hippocampus. The findings point to an association of local Aβ-driven and immune-mediated responses with altered L-arginine metabolism, and they suggest that arginase and S6K1 inhibition by L-norvaline may delay the progression of AD.

**Electronic supplementary material:**

The online version of this article (10.1007/s13311-018-0669-5) contains supplementary material, which is available to authorized users.

## Introduction

Alzheimer’s disease (AD) is a slowly progressive neurodegenerative disorder, with an insidious onset. Advanced age is a prominent risk factor for AD, atherosclerosis, and metabolic disorders, such as type II diabetes. Their causal mechanisms are multifaceted and not fully interpreted [1]. Recent clinical and experimental data have shown that neurodegenerative disorders often coexist with metabolic dysfunction [2]. According to 1 recent hypothesis, impaired bioenergetic metabolism may play a key role in the pathogenesis of AD [3]. This hypothesis proposes that AD is characterized by a combination of several interrelating pathological events, which include bioenergetic, metabolic, neurovascular, and inflammatory processes. A recent study provided evidence that brain hypo-metabolism occurs decades before clinical manifestations of AD, further suggesting that metabolic dysfunction is a causal factor in AD [4].

New objective lipidomics and metabolomics approaches have been applied to analyze changes in postmortem brains of AD patients [5]. These approaches have strongly implicated the L-arginine metabolic pathway in the development of AD [6]. Similar results pointing to dysregulation of L-arginine metabolism were acquired in a rodent model of AD [7].

In initial clinical studies on L-arginine and its derivatives in patients with various neurological disorders, L-arginine administration within 30 min of a stroke significantly decreased the frequency and severity of stroke-like symptoms [8]. Supplementation with 1.6 g/day of L-arginine for 3 months in patients with senile dementia increased cognitive function by about 40% [9]. A recent study also established the benefit of L-arginine administration in a rodent model of AD [10].

In healthy individuals, L-arginine is transported from the circulating blood into the brain via a Na^+^-independent cationic amino acid transporter 1 (CAT1), which is expressed at the blood-brain barrier (BBB) and functions as a supply pathway for L-arginine to the brain [11, 12]. In mammals, L-arginine is derived mostly from renal *de novo* synthesis and dietary intake. Thus, despite the capability of the CAT1 to pass through the BBB, its capacity as a transporter of L-arginine is limited [13]. As a result, oral administration is not an option to induce the potential neurotrophic benefits of L-arginine. However, pharmacological targeting of enzymes that metabolize L-arginine may be a beneficial method to treat neurological disorders, such as AD.

Recent research has pointed to a role for arginase, the enzyme that converts L-arginine to urea and L-ornithine, in AD, cardiovascular diseases, and metabolic disorders [14]. One study detected significantly decreased levels of L-arginine in the cortices of AD patients [15]. In addition, the activity of arginase was significantly higher in the hippocampi of AD patients [16]. Consequently, a hypothesis linking L-arginine brain deprivation and the development of AD cognitive deficiency was proposed [17].

There are 2 arginase enzymes, arginase I (ARGI) and arginase II (ARGII). ARGI and ARGII are both expressed in the rodent brain, especially in hippocampal neurons [18]. Previous research demonstrated that ARGII was the predominant isoform in the human frontal cortex [19]. The expression of both isoforms of arginase can be induced in different tissues via exposure to a variety of cytokines and catecholamines [20]. Stimuli that induce ARGII and lead to its translocation from mitochondria to the cytosol include lipopolysaccharides, tumor necrosis factor-α, oxidized low-density lipoprotein, and hypoxia [21, 22].

A recent study provided evidence for increased ARGII gene expression in AD brains [23]. Studies also demonstrated that ARGII deficiency reduced the level of hyperoxia-mediated retinal neurodegeneration [24] and suggested that arginase may be involved in the pathogenesis of neuronal degeneration via excessive activation of excitotoxic *N*-methyl-D-aspartate receptors (NMDARs) [25]. Therefore, targeting ARGII has been proposed as a potential treatment for decelerating age-related diseases [26].

Another way in which L-arginine may confer neuroprotection is through its effects on the nitric oxide (NO) pathway. Nitric oxide synthase (NOS) utilizes L-arginine as a substrate to produce NO and L-citrulline [27]. Accordingly, the bioavailability of L-arginine influences NO synthesis [28]. A growing body of evidence indicates that NO induces neuroprotection by triggering vasodilation and increasing the blood supply to neurons, thereby reducing their susceptibility to oxidative stress [29]. Moreover, NO regulates Ca^2+^ influx into neurons and provides neuroprotection against excitotoxicity [30]. A previous study demonstrated reduced NOS activity in AD brains, with a decrease in the levels of NOS1 and NOS3 proteins [16].

Previously, an APP/NOS2^−/−^ murine model was developed to investigate the role of NO in the development of AD [31]. These mice exhibit behavioral deficits and biochemical hallmarks of AD during aging. In this murine model, ARGI was highly expressed in regions of amyloid-beta (Aβ) accumulation [17]. Pharmacological disruption of the arginine utilization pathway by irreversible inhibition of ornithine decarboxylase with α-difluoromethylornithine (DFMO) protected the mice from AD-like pathology and reversed memory loss. The authors of the study suggested that L-arginine depletion was responsible for neuronal cell death and cognitive deficits in the course of AD development.

In another study, the arginase inhibitor L-norvaline amplified the level of NO production and reduced urea production [31]. L-norvaline, which exerts its activity via negative feedback regulation, was later used successfully to treat artificial metabolic syndrome in a rat model [32]. Research also showed that L-norvaline inhibited the activity of ribosomal protein S6 kinase β-1 (S6K1) and that it possessed anti-inflammatory properties [33], indicating that L-norvaline could be extremely effective in AD.

Based on the literature, we hypothesized that upregulation of arginase activity and consequent L-arginine and NO deficiencies in the brain might contribute to the manifestations of AD pathology and that targeting arginase might ameliorate the symptoms of the disease.

In this study, we treated triple-transgenic (3×Tg) mice with L-norvaline dissolved in water. Animals from a control group displayed significant memory deficiencies as compared with those in the treated (experimental) group, as evident from 2 paradigms. The cognitive improvements observed in the treated group were associated with reduced levels of beta-amyloidosis, microgliosis, and astrodegeneration. The L-norvaline treatment also reversed the decline in dendritic spine density in the hippocampi and amplified the expression levels of pre- and postsynaptic proteins.

## Methods

### Strains of Mice and Treatment

Homozygous 3×Tg mice harboring a mutant APP (KM670/671NL), which is a human mutant PS1 (M146V) knock-in, and tau (P301L) transgenes (B6;129-Psen1^tm1Mpm^ Tg(APPSwe, tauP301L)1Lfa/J) were obtained from Jackson Laboratory (Bar Harbor, ME) and bred in our animal facility. These mice exhibit a synaptic deficiency, with both plaque and tangle pathology [34]. Four-month-old homozygous 3×Tg mice and age-matched male C57BL/6 mice were used as nontransgenic controls (non-Tg) in all the experiments. C57BL/6 mice are commonly used as non-Tg controls of 3×Tg mice [35]. The average weights of the 3×Tg and non-Tg animals were marginally different at the beginning of the experiment. However, the difference was not statistically significantly different, and the difference was negligible at the end of the experiment.

Randomly chosen animals were divided into 4 groups (14–15 mice in each group). The animals were housed in cages and provided with water and food ad libitum. The dose administered was about 40–50 mg/kg/day, which was similar to that employed in a previous study in which L-norvaline was used to treat artificial metabolic syndrome in a rat model [32]. In the earlier study, the animals received norvaline dissolved in water at a dose of 50 mg/kg/day for 6 week via force gavage feeding. In the present study, L-norvaline (Sigma) was dissolved in the animals’ water (250 mg/L) supplied in the animals’ cages. For 3×Tg mice aged 5 months with an average weight of about 30 g (33 g by the end of the experiment), the dose was about 1.5 mg/day, taking into account that the average consumption of water by mice under laboratory conditions equals about 5–6 mL/day [36].

**Fig. 1:**
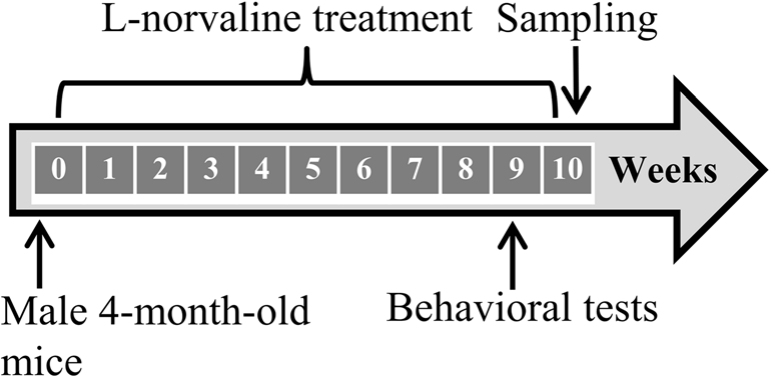
Experimental design

The mice underwent appropriate treatment. The experimental design is presented in Fig. 1. The weights of the animals were measured every week during the experiment, and no significant treatment-related effects on the weights were observed.

All animal housing and procedures were performed in compliance with the guidelines established by the Israeli Ministry of Health’s Council for Experimentation on Animals and with Bar-Ilan University guidelines for the use and care of laboratory animals in research. The experimental protocol was approved by the ethics committee for animal experiments of Bar-Ilan University (Permit Number: 82-10–2017).

### Behavioral Tests

#### Morris Water Maze

The Morris water maze (MWM) task was conducted to measure long-term learning and memory function as described previously [37]. Briefly, a black plastic pool with a diameter of 120 cm and a height of 50 cm was filled with water (24 ± 2 °C) and rendered opaque by the addition of skim milk powder (Sigma). A circular dark colored platform with a diameter of 10 cm was placed 0.3 cm under the water surface in 1 of the quadrants. It remained in this quadrant throughout the tests. Training was conducted on consecutive days for 7 days, with 2 trials each day.

The test consisted of 3 separate procedures. On the first day, the platform was visible during the test, and a flag was placed on the platform to increase its visibility [38]. The visible platform test aimed to identify gross visual deficits caused by the treatment that could confuse subsequent interpretations of the results obtained in the MWM experiment [37]. The mice underwent 2 trials with 90-s cutoffs each and a 15-min interval between the trials. On days 2–6, the tests were conducted with the water platform hidden and no flag. Each animal was placed in the pool at 1 of 4 locations (poles). The mice were required to find the platform within 90 s, and they were allowed to remain on the platform for 15 s. If a mouse failed to locate the platform during the time limit, it was directed gently toward the platform. A probe test without the platform was performed on the 7th day. In this test, the mice were released into the center of the maze and subjected to a single trial with free swimming for 90 s.

#### Spontaneous Alternation Y-Maze

Short-term working memory was assessed in a Y-maze consisting of 3 arms (each 35 cm long, 25 cm high, and 10 cm wide) at a 120° angle from each other. After an introduction to the center of the maze, each animal was allowed to freely explore the maze for 8 min. The maze was cleaned with a 10% aqueous ethanol solution between each trial. Spontaneous alternation was defined as successive entry into 3 different arms on overlapping triplet sets. An arm entry was counted when the hind paws of the mouse were entirely within the arm [39].

#### Video Tracking

All the behavioral experiments were recorded using a Panasonic WV-CL930 camera with a Ganz IR 50/50 infrared panel. The recorded video files were analyzed using Ethovision XT 10 software (Noldus Information Technology, Wageningen, Netherlands) by an individual blinded to the treatment schedule.

### Tissue Sampling

After the behavioral tests, the mice were deeply anesthetized with sodium pentobarbital (60 mg/kg) administered intraperitoneally and decapitated. Their brains (4 from each group) were sliced (0.5 mm thick) immediately in a mouse brain slicer matrix. Sections between 1.7 and 2.2 mm posterior to bregma according to the atlas of Franklin and Paxinos were used for sampling [40]. The hippocampi were punched in the region of the dentate gyrus in each hemisphere using a 13-gauge microdissection needle, frozen, and stored at − 80 °C.

### Antibody Microarray

The assessment of “hit” proteins’ expression was performed using a Kinex KAM-1150E antibody microarray (Kinexus Bioinformatics) in accordance with the manufacturer’s instructions. The analyses were done with hippocampal lysates as described on Kinexus’ web page (www.kinexus.ca). Briefly, lysate protein from each sample (100 μg) was labeled covalently with a fluorescent dye combination. Free dye molecules were then removed via gel filtration. After blocking nonspecific binding sites on the array, an incubation chamber was mounted onto the microarray to permit the loading of the samples. After incubation, unbound proteins were washed away. Two 16-bit images from each array were then captured using a ScanArray Reader (Perkin-Elmer). An antibody array was performed in parallel on 4 different chips. The output of the array consisted of the average normalized net signals (i.e., the average of 4 normalized net signal values of each antibody on the microarray).

The standard deviation and percent standard deviation of 4 separate measurements of globally normalized signal intensity values for each different antibody on the microarray were calculated. The data are presented as change from control (CFC%). A positive value corresponded to an increase in signal intensity in response to the treatment, with a value of 100% corresponding to a 2-fold increment in signal intensity. A negative CFC value indicated the degree of reduction in signal intensity from that of the control.

Each parameter has its Student’s *t* test *p* value, which is the probability (*p*) value that there is no difference between the control and test samples. A *p* value determined with *N* = 4 measurements in each set, which were paired and 2-tailed in distribution. A *p* value of ≤ 0.05 was accepted as statistically significant.

### Golgi Staining Procedure

For Golgi staining, 3 mice in each group were perfused transcardially with 0.1 M phosphate-buffered saline (pH 7.4), and their brains were processed using a superGolgi Kit according to the manufacturer’s protocol (Bioenno Lifesciences, Santa Ana, CA, USA). Briefly, the brains were immersed in impregnation solution for 11 days, followed by incubation for 2 days in a postimpregnation solution. Once the impregnation of neurons was complete, the brain samples were coronally sliced (150 μm) using a vibrating microtome (Campden Instruments, Lafayette, IN) and collected serially in a mounting buffer. They were then mounted upon 1% gelatin-coated glass slides, stained with a staining solution, and coverslipped using Permount (Fisher Scientific, Houston, TX, USA). Two corresponding sections between 1.7 and 2.0 mm posterior to bregma according to the atlas of Franklin and Paxinos per animal were chosen for stereological analysis [40].

An upright ApoTome (Quorum Technologies, Lewes, UK) microscope was used for imaging. To assess dendritic morphology, low magnification (× 10/0.3, × 20/0.8, and × 40/0.75 lens) images (Z-stack with 0.5 μm intervals) of pyramidal neurons, with cell bodies located in the CA1 region of the hippocampus, were captured using an ORCA-Flash4.0 V3 camera. High-magnification (× 100/1.4 oil objective with digital zoom 3) images (Z-stack with 0.25 μm intervals) were also obtained.

### Dendritic Spine Density Measurement

The captured images were coded, and an investigator blinded to the experimental conditions measured the numbers of spines per dendritic length using Zen Blue 2.5 (Zeiss, Oberkochen, Germany) and Neurolucida 360, version 2018.1.1 (MBF Bioscience, Williston, VT, USA). software, which provides an automatic unbiased quantitative 3D analysis of identified neurons [41]. The spine detection threshold was set an outer range of 2.5 μm, the minimal height was 0.3 μm, the sensitivity was 100%, and the minimum count was 10 voxels.

The spine density was analyzed in each hemisphere of each murine brain. Two hippocampal pyramidal cells, with soma located in the center of 150-μm corresponding sections, were selected for the analysis (24 neurons per experimental group). The spine density on a secondary oblique dendritic branch localized in the stratum radiatum of the CA1 hippocampal pyramidal neurons was also quantified.

### Immunostaining

Seven animals from each group were deeply anesthetized and transcardially perfused with 30 mL of phosphate-buffered saline (PBS), followed by 50 mL of chilled paraformaldehyde 4% in PBS. The brains were carefully removed and fixed in 4% paraformaldehyde for 24 h. The brains from 3 mice per group were then transferred to 30% sucrose in PBS at 4 °C for 24 h and frozen at − 80 °C. The brains from another 4 mice (including non-Tg mice) in each group were dehydrated and paraffin embedded.

### β-Amyloid, 1-16 (6E10) Antibody Staining

The brains were sliced on a Leica (Wetzlar, Germany) CM3050 S cryostat to produce 30-μm floating sections. For β-amyloid, 1-16 (6E10) antibody staining, corresponding sections (between 1.7 and 2.2 mm posterior to bregma) were blocked and incubated overnight with primary purified anti-β-amyloid, 1-16 antibody 6E10 (1:100, Biolegend) at 4 °C. This was followed by washing and incubation with goat anti-mouse Immunoglobulin G secondary antibody Alexa555 (1:200, Invitrogen) at room temperature for 1 h and Hoechst (1:5000, Sigma) for 3 min.

### Quantitative Histochemical Analysis of Microglia, Astrocytes, ARGI, and ARGII

The paraffin-embedded tissue blocks were chilled on ice and sliced on a Leica (Leica Biosystems, Nussloch, Germany) RM2235 manual rotary microtome at a thickness of 4 μm. The sections were mounted onto gelatin-coated slides, dried overnight at room temperature, and stored at 4 °C inside storage boxes.

For the quantitative histochemical analysis of microglia (Iba1), astrocytes (GFAP), ARGI, and ARGII immunopositivity, 5 brain coronal sections per mouse (1.9–2.0 mm posterior to bregma) were used. Five serial sections at a thickness of 4 μm were cut at 20-μm intervals throughout the brain. Immunohistochemistry (IHC) was carried out on the plane-matched coronal sections.

Staining was performed on a fully automated Leica Bond III system (Leica Biosystems Newcastle Ltd., UK). The tissues were pretreated with an epitope-retrieval solution (Leica Biosystems Newcastle Ltd., UK), followed by incubation with primary antibodies for 30 min. The Iba1 antibody (Novus Biologicals, #NB100-1028), GFAP (Biolegend, #835301), ARGI (GeneTex, GTX113131), and ARGII (GeneTex, #GTX104036) dilutions were 1:500, 1:1000, 1:500, and 1:60, respectively. A Leica Refine-HRP kit (Leica Biosystems Newcastle Ltd., UK) was used for detection and counterstaining with hematoxylin. The omission of Iba1, GFAP, ARGI, and ARGII primary antibodies served as a negative control (Supplementary Fig. S1). For a positive control of ARGI, murine liver tissue and kidney were stained for ARGII.

### Imaging and Quantification

The sections were mounted and viewed under an Axio Scan.Z1 (Zeiss, Oberkochen, Germany) fluorescent and bright-field slide scanner with a × 40/0.95 objective. Images were taken with a Z-stack of 0.5 μm. Also, an Axio Imager 2 Upright ApoTome microscope was used to capture images with a × 100/1.4 oil immersion objective. Immunolabeling was performed in the corresponding hippocampal areas, and image analysis was carried out using Zen Blue 2.5 (Zeiss, Oberkochen, Germany) and Image-Pro® 10 (Media Cybernetics, Rockville, MD) software with a fixed background intensity threshold for all sections representing a single type of staining.

### Morphometric Cell Analysis

Resting microglia exhibit a ramified phenotype and adopt different morphologies, ranging from a highly ramified to an amoeboid-like phenotype upon activation [42]. To quantify morphological changes in resting microglia, we used morphological parameters that are accepted in the literature and expected to capture the shift from resting to activated phenotype [43]. The parameters included the cell perimeter length, Feret’s diameter, soma size, and sphericity (4π × area/perimeter^2^). To assess the staining density, the integrated optical density (IOD), which was defined as the mean density per pixel area (lum/pix^2^), was calculated [44]. The IOD values of the Iba1-stained microglia from CA3 regions with a surface area of 0.1 mm^2^ were calculated as log_10_ of the values acquired using Image-pro® 10. Two appropriate sections (bregma − 1.8 and − 2.0 mm) from each mouse were included in the analyses (*N* = 125 cells in the untreated groups; *N* = 90 cells in the L-norvaline-treated groups).

### Western Blotting

To determine the level of β-amyloidosis, the immunoreactivity of the A11 antibody and anti-amyloid fibril OC antibody in hippocampal lysates was examined. The A11 antibody detects the conformation of amyloid oligomers, irrespective of their amino acid sequence [45], and the anti-amyloid fibril OC antibody recognizes fibrillary forms.

In addition, the relative differences in the expression levels of various neuroplasticity-related proteins that showed significant changes in the antibody microarray assay were analyzed and validated. The setup and the antibody nomenclature are presented in Supplementary Table 1.

The protein concentration was determined using the Bradford assay. Hippocampal lysates (15 μg) obtained from brain samples of the 3×Tg mice treated with L-norvaline or vehicle (4 tissue punches from each group) were analyzed using a Kinetworks™ Custom Multi-Antibody screen 1.0 (Kinexus Bioinformatics, Vancouver, Canada) in accordance with the instructions of the manufacturer. Briefly, the Kinetworks™ analysis involves resolution of a single lysate sample by sodium dodecyl sulfate-polyacrylamide gel electrophoresis and subsequent immunoblotting using validated antibodies. Antibodies bound to their target antigen on the nitrocellulose membrane were detected using an enhanced chemiluminescence detection system.

### Statistical Analysis

Statistical analyses were conducted using SPSS version 22 (IBM, Armonk, NY, USA) for Windows. The significance was set at 95% of confidence. All the results are presented as mean with standard error. The Shapiro–Wilk test showed that the data fit a normal distribution, and Levene’s test was performed to confirm equal variance between the groups being compared.

For the Y-maze, visible platform test, and probe trials (MWM), the means were compared between groups using a one-way analysis of variance (ANOVA), with Tukey’s multiple comparison test used for post hoc analyses. For the hidden platform test, the escape latencies in the 2 trials were averaged for each mouse each day and then analyzed across the 5 days of testing [37]. An ANOVA was applied, with day as the repeated measure and latency as the dependent variable. For comparisons between groups, Tukey’s multiple comparison test was conducted when the main analysis revealed significance. The Student *t* test was performed to compare the means between 2 groups. All data are presented as mean values. Throughout the text and in bar plots, the variability is indicated by the standard error of the mean (SEM). In the figures with box and whisker graphs, the boxes extend from the 25th to 75th percentiles. The line in the middle of the box is plotted at the median. The whiskers denote the smallest and largest values.

## Results

### L-Norvaline Ameliorated Memory Deficits in the 3×Tg Mice

To investigate whether L-norvaline treatment affected learning and memory in AD pathogenesis, a set of behavioral tests was administered following L-norvaline treatment for 2 months, beginning when the mice were aged 4 months.

**Fig. 2:**
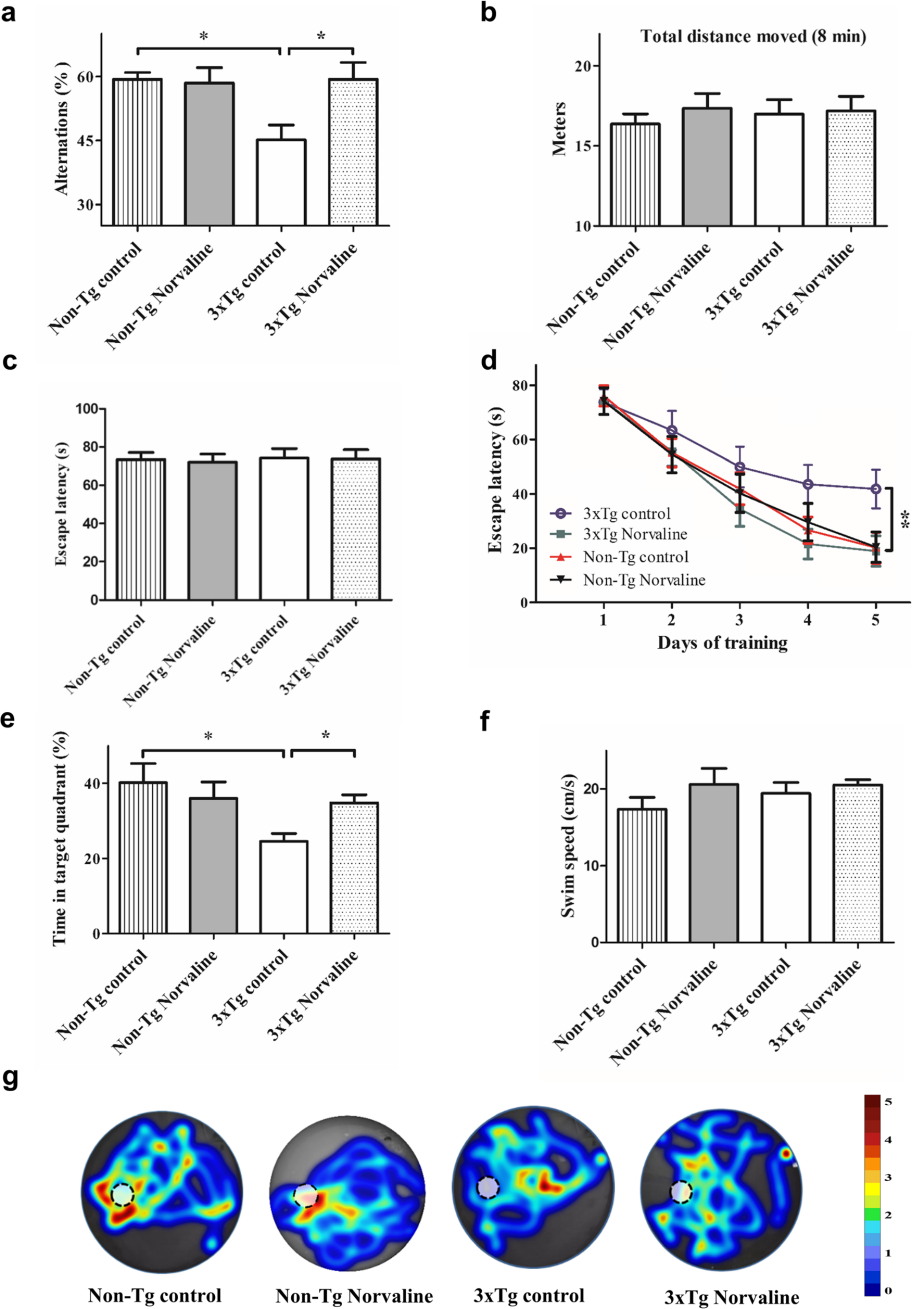
The effects of L-norvaline on animal behavior. The Y-maze spontaneous alternation test. **(a)** Percentage of alternation. **(b)** The total distance traveled during a trial. **(c)** (MWM) The visible platform test (averaged escape latencies of 2 trials). **(d)** The hidden platform test (averaged escape latencies of 2 trials/day). **(e)** The time spent by the mice in the target quadrant in the probe trial. **(f)** The average swimming speed in the probe test. **(g)** Heat maps showing the search intensity during the probe trials, where a dashed circle indicates the platform. A high dwell time across the pool area is indicated by colors close to red, whereas colors close to blue indicate lower dwell time (arbitrary scale). Data are shown as means ± SEM. (*n* = 15 in the non-TG groups, *n* = 14 in the 3×Tg groups). *p < 0.05, **p < 0.01

Short-term working memory was assessed in a Y-maze spontaneous alternation test. The results of the one-way ANOVA test revealed a significant treatment-related effect on the “percentage of alternations” (*F*(3,54) = 4.525, *p* = 0.0067). The post hoc Tukey multiple comparison test indicated that the percentage of alterations in the control 3×Tg mice was lower than that in the L-norvaline-treated mice and wild-type (WT) controls (*p* < 0.05) (Fig. 2a). The L-norvaline treatment had no significant effect on the alternative behavior of the non-Tg mice. The treatment also did not affect the total distance traveled by the animals during the test (*p* = 0.86) (Fig. 2b).

The MWM was used to determine the effect of L-norvaline upon spatial memory. In the visible platform test, there were no significant between-group differences (*p* = 0.99), which indicated that the treatment had no influence on murine motility or vision (Fig. 2c).

In the hidden platform swimming test, there were significant differences between the groups according to the repeated measures ANOVA (*F*(3,12) = 7.725, *p* = 0.0039). Tukey’s multiple comparison test revealed a significant (*p* < 0.01) effect of the treatment on memory acquisition in the 3×Tg group (Fig. 2d).

In the probe trial on the last day of testing, the platform was removed. The relative time spent by the mice in the target quadrant, where the hidden platform had been previously placed, was significantly longer in the L-norvaline-treated group as compared with that of the control group (34.8 ± 8.06 s *vs* 24.5 ± 7.88 s; *p* < 0.05; Fig. 2e). To visualize spatial preferences in the probe test, a heat map was used (Fig. 2g). The plots demonstrated that the treated 3×Tg animals spent more time than the control mice did in the target quadrant. The treatment did not affect the swim speed of the animals (Fig. 2f).

Overall, the results of the behavioral experiments suggested that L-norvaline improved spatial memory acquisition in 3×Tg mice.

### The L-Norvaline Treatment Reduced the Quantities of Aβ Fibrils and Prefibrillar Oligomers in the Hippocampi of the 3×Tg Mice

Recent evidence pointed to a role for soluble amyloid oligomeric and fibrillar species in synaptic dysfunction, neuronal apoptosis, and brain damage [46]. To investigate the impact of L-norvaline on the amount of toxic prefibrillar Aβ oligomers and fibrillary forms, we examined A11 and OC immunoreactivity of hippocampal lysates. Pooled equal amounts (10 μg) of total lysates from the hippocampi of each group of mice were run on sodium dodecyl sulfate–polyacrylamide gels and incubated with appropriate antibodies (Supplementary Table 1 and Supplementary Fig. S2). The L-norvaline treatment resulted in a substantial (about 30%) reduction in the levels of A11-reactive oligomers and OC-reactive fibrillar species (Table 1).

**Table 1:** L-Norvaline reduced the level of fibrillar amyloid and prefibrillar oligomers in hippocampal brain lysates of the 3×Tg mice (blots’ summary). Western blot, showing β-actin normalized trace quantities of Aβ fibrils (a) and prefibrillar oligomers (b)

(a) Actin normalized trace quantity				(b) Actin normalized trace quantity				
MW (kda)	Control	Norvaline	Change (%)	MW (kda)	Control	Norvaline	Change (%)	Structure
135.938	514	265	− 48	85.776	787	141	− 82	
89.692	428	518	+ 21	61.237	1622	1288	− 22	
79.654	574	308	− 46	48.587	8723	6127	− 30	Dodecamers
68.494	590	521	− 12	40.324	150	30	− 80	Octamers
62.231	725	593	− 18	36.076	393	456	+ 16	
50.635	1703	1206	− 30	33.865	406	462	+ 14	
43.94	1799	1185	− 34	27.314	2784	1502	− 46	Hexamers
37.763	1150	1289	+ 12	24.216	1218	1245	+ 2	
25.766	4254	3229	− 24					
Sum	11,737	9114	− 22	Sum	16,084	11,251	− 30	

### L-Norvaline Decreased the Level of β-Amyloidosis in the Cortex of the 3×Tg Mice

**Fig. 3:**
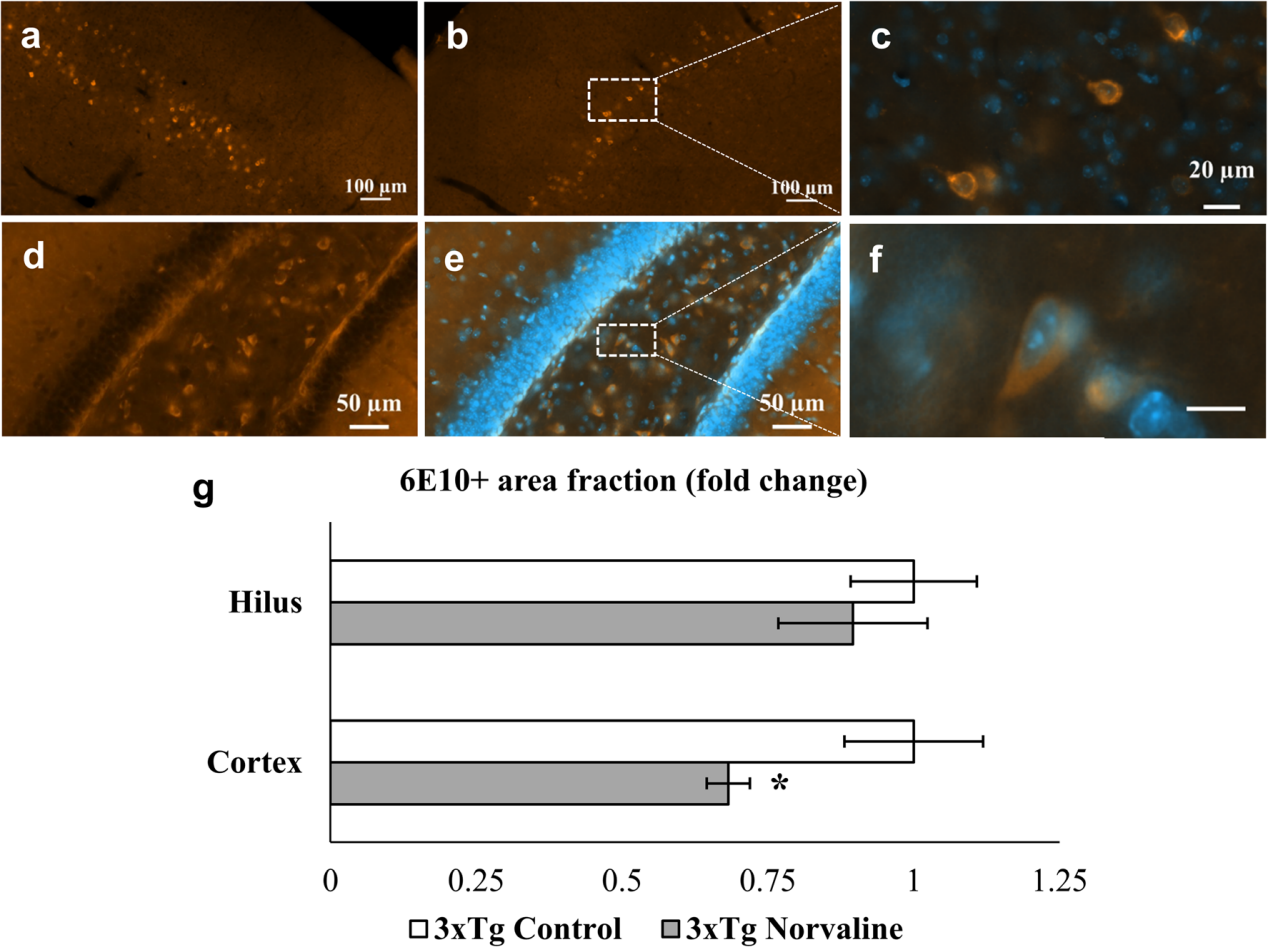
The impact of L-norvaline on the total amyloid burden. Quantification of the area of Aβ immunoreactivity in brain sections from the 3×Tg mice treated with vehicle (control) or L-norvaline. Representative × 20 magnification view of PFC from the control **(a)** and L-norvaline-treated mice **(b)** with apparent deposits in layers IV–V. The insets **(c**, **f)** represent × 100 magnification of double staining with DAPI. Intense Aβ deposition in the dentate gyrus **(d**–**f)** of the 3×Tg control mice, (**f**, scale bar 20 μm). Bar chart of the immunoreactive area in the hilus and PFC **(g)**. The Student *t* test was used to compare the means between 2 groups, *p < 0.05 (*n* = 15, 3 mice per group)

To examine the effect of the L-norvaline treatment on the total amyloid burden in the brains of the 3×Tg mice, coronal brain sections were stained with human APP/Aβ-specific antibody. By the time they were 6 months old, the 3×Tg mice exhibited enhanced intracellular deposition of Aβ in the IV–V layers of the prefrontal cortex (PFC) (Fig. 3a–c) and hippocampus (Fig. 3d–f).

We did not detect significant differences in the level of Aβ deposition in the hippocampi of the 2 groups. However, there was a general trend toward a slight reduction in 6E10 positivity in the treated group. There was a significant (*p* = 0.032) reduction in the level of 6E10 positivity in the cortex of the 3×Tg mice treated with L-norvaline (Fig. 3g). These data pointed to an effect of L-norvaline on the level of β-amyloidosis in the 3×Tg mice.

### L-Norvaline Increased Pyramidal Cell Dendritic Spine Density in the Hippocampus of the 3×Tg Mice

Dendritic spines are specialized structures on neuronal processes, which are involved in neuronal plasticity [47]. A strong correlation between dendritic spine density and memory acquisition was demonstrated in rodents using different behavioral paradigms [48]. Furthermore, 3×Tg mice showed progressive dendritic spine deficiency as compared with WT mice [49], and this deficiency can be reversed by the treatments [50].

**Fig. 4:**
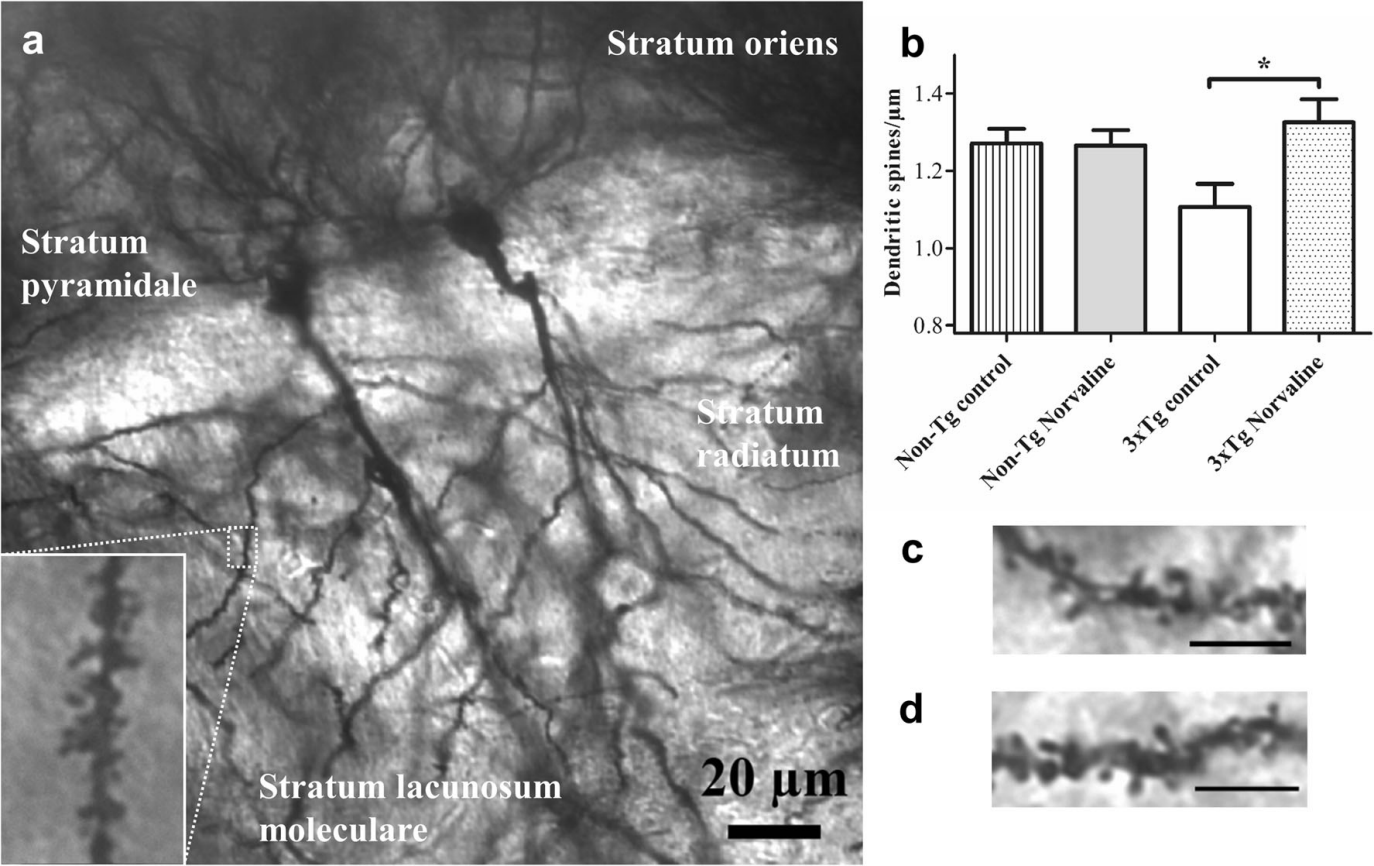
Photomicrographs of Golgi-stained hippocampal neurons from a coronal slice. **(a)** A representative Golgi-impregnated × 40 image of the CA1 region of non-Tg mice, showing 2 pyramidal cells. The inset is a × 100 image of a secondary apical oblique dendrite in the stratum radiatum. Automated quantitation of spine density using Neurolucida software was subjected to a statistical analysis by a one-way ANOVA. **(b)** Spine density of hippocampal neurons. *p < 0.05. The data are mean ± SEM, *n* = 24 for hippocampal cells (3 mice per group). Representative dendritic CA1 segments of 3×Tg control **(c)** and 3×Tg L-norvaline-treated mice **(d)**. The scale bar is 5 μm

In the present study, dendritic spines were examined by Golgi staining in the vehicle- and L-norvaline-treated groups. Dendritic spine density was quantified on a secondary oblique dendritic branch localized in the stratum radiatum of the CA1 hippocampal pyramidal neurons (Fig. 4a, c, d). The results of the one-way ANOVA test revealed a significant effect of the treatment on dendritic spine density (*F*(3,92) = 3.338, *p* = 0.0173). The post hoc Tukey multiple comparison test pointed to a significantly lower spine density in the control 3×Tg mice as compared with that in the L-norvaline-treated mice (*p* < 0.05) (Fig. 4b). The average increase following the treatment was about 20% (1.326 ± 0.059 *vs* 1.10767 ± 0.06 spines per micrometer). The treatment did not affect dendritic spine density in the non-Tg groups.

### L-Norvaline Amplified the Expression Levels of Proteins Related to Synaptic Plasticity in the 3×Tg Mice

**Fig. 5:**
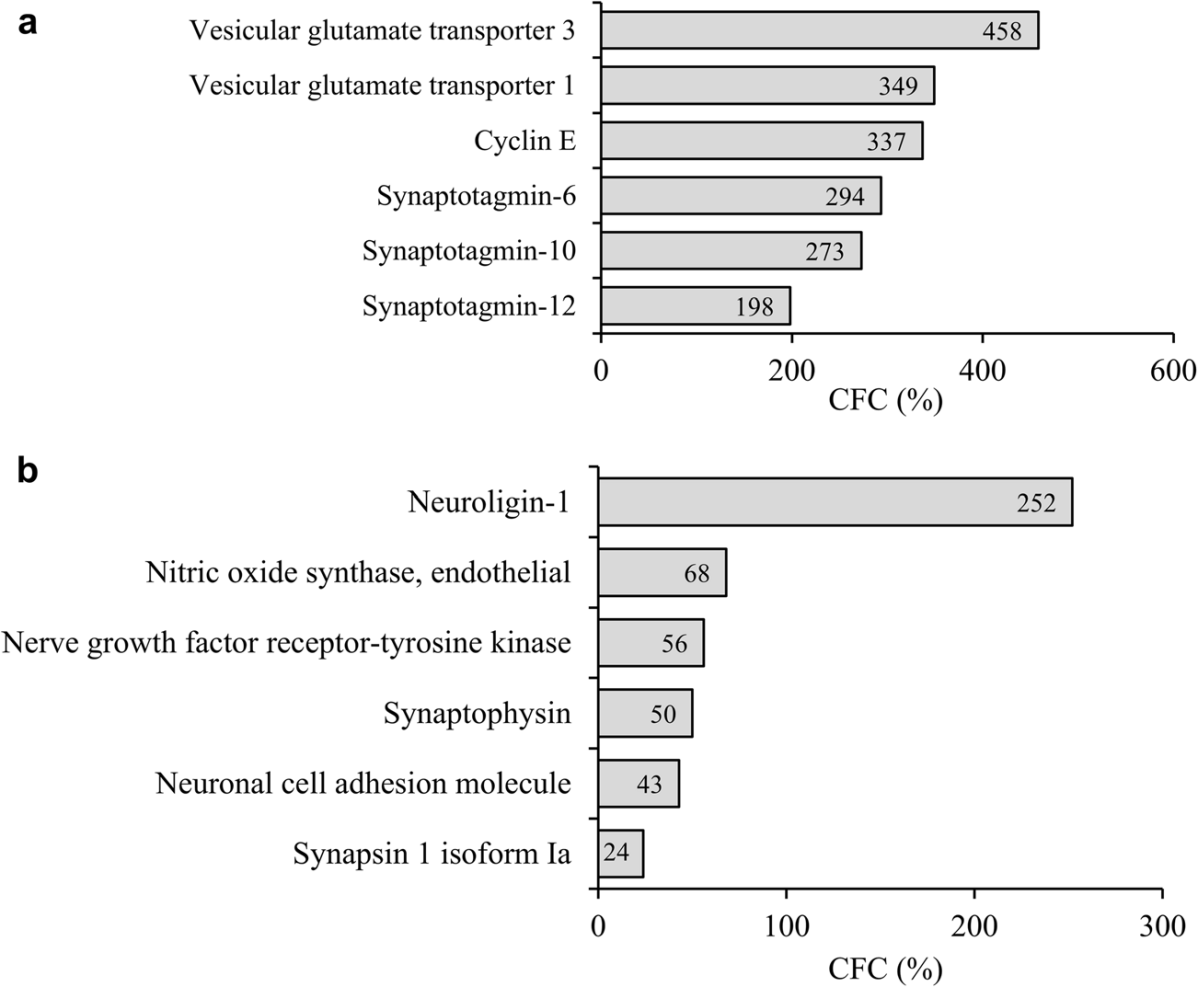
L-Norvaline increased the expression levels of neuroplasticity-related proteins in the hippocampus of the 3×Tg mice. Results of the Western blot with β-actin normalized trace quantities **(a)** and antibody array selected data **(b)**. CFC = change from control

To gain additional molecular insight into the behavioral phenotype, the effect of the L-norvaline treatment on the levels of neuroplasticity-associated proteins in the 3×Tg mice was investigated using a set of proteomics assays. First, we tested the protein levels of 12 known important neuroplasticity-related proteins in the hippocampus by a Western blot analysis (Supplementary Table 1). Second, we performed an antibody array analysis to detect changes in protein levels at the whole throughput level (approximately 1000 proteins). The Western blot analysis revealed an increase in synaptic- and neuroplasticity-related proteins in the L-norvaline-treated group (Fig. 5a). Remarkably, the expression of vesicular glutamate transporters 1 and 3 in the brain increased by 458% and 349% respectively, potentially increasing vesicular glutamate levels.

The protein microarray analysis revealed elevated levels of other neuroplasticity-related proteins following the L-norvaline treatment. The expression level of postsynaptic protein neuroligin-1, which mediates the formation and maintenance of synapses, was amplified by 252% following the treatment. However, the *p* value of the change detected by the antibody microarray was 0.07. Furthermore, the levels of synaptophysin, which is a synaptic vesicle glycoprotein, increased by 50% in the hippocampi of the 3×Tg mice treated with L-norvaline, and this finding was statistically significant (*p* = 0.039).

Notably, the expression of endothelial NOS increased by 68% (*p* = 0.041). Moreover, there was a substantial significant (*p* = 0.015) increase in cyclin E protein, which is a central component of the cell cycle machinery and known to regulate synaptic plasticity and memory formation [51].

### L-Norvaline Reduced Microgliosis in the 3×Tg Mice

**Fig. 6:**
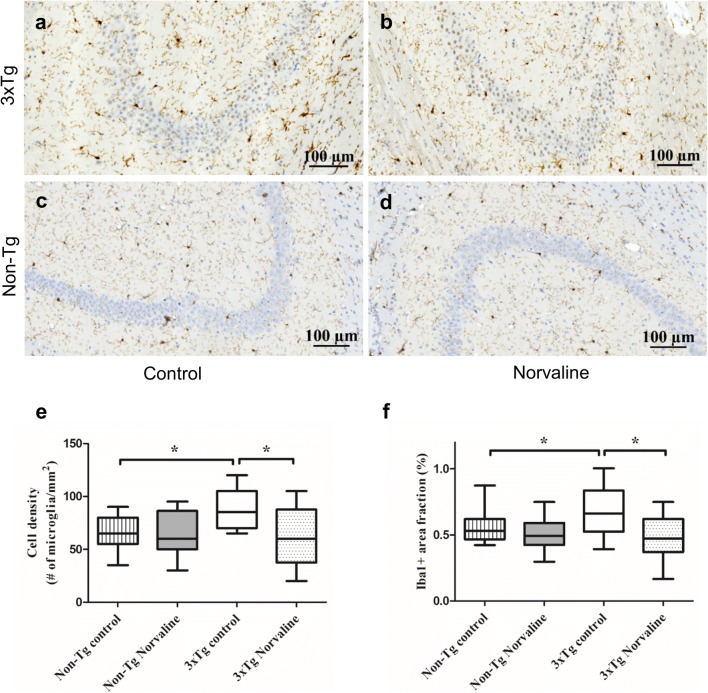
L-Norvaline significantly decreased the microglial density in the 3×Tg mice. Visualization and quantification of microglia with Iba1 staining of the hippocampus of the 3×Tg mice **(a**, **b)** and non-Tg mice **(c**, **d)**. Representative hippocampal × 20 bright-field micrographs of the CA3 areas of the control **(a**, **c)** and L-norvaline-treated mice **(b**, **d)**. The boxplots show the area density of microglia in the hippocampal CA3 area of the 3×Tg mice and non-Tg mice treated with vehicle or L-norvaline **(e)**. A significant reduction in the area fraction (%) of Iba1 immunoreactivity in the hippocampal CA3 areas of the 3×Tg mice treated with L-norvaline **(f)**. *p < 0.05 *versus* controls using a one-way ANOVA with Tukey’s post hoc test, *n* = 20, 4 mice per group

Microglia are the first line of active immune defense in the brain, and an increase in microglial density is indicative of elevated pathogenic insults [52]. Previous studies reported increased microglial density in the hippocampi of AD patients [53] and hippocampi of 3×Tg mice [54]. Microglia densities in the CA3 area (0.2 mm^2^) of the 3×Tg (Fig. 6a, b) and non-Tg (Fig. 6c, d) mice were analyzed using image-processing software and a manually set threshold. To guarantee the selection of only cells present in the acquired field, for Iba1 staining, only cells with an area greater than 25 μm^2^ were considered in the analysis, as widely accepted in the literature [55].

The results of the one-way ANOVA test revealed a significant effect of the treatment on microglial density and the “Iba1-positive area fraction” (*F*(3,52) = 4.64, *p* = 0.006 and *F*(3,52) = 4.098, *p* = 0.01, respectively). The post hoc Tukey multiple comparison test indicated a significant reduction (*p* < 0.05) in microglial density in the area of interest in the L-norvaline-treated 3×Tg group (63.84 ± 7.43 *vs* 83.66 ± 5.11 cells/mm^2^, on average) (Fig. 6e) and a significant (*p* < 0.05) decrease in the Iba1-positive area fraction (0.48 ± 0.04 *vs* 0.67 ± 0.05%) (Fig. 6f).

### The Microglial Morphology of Iba1-Positive Cells in the Hippocampus of the L-Norvaline-Treated 3×Tg Mice Pointed to a Prevalence of the Resting (Ramified) Phenotype

**Fig. 7:**
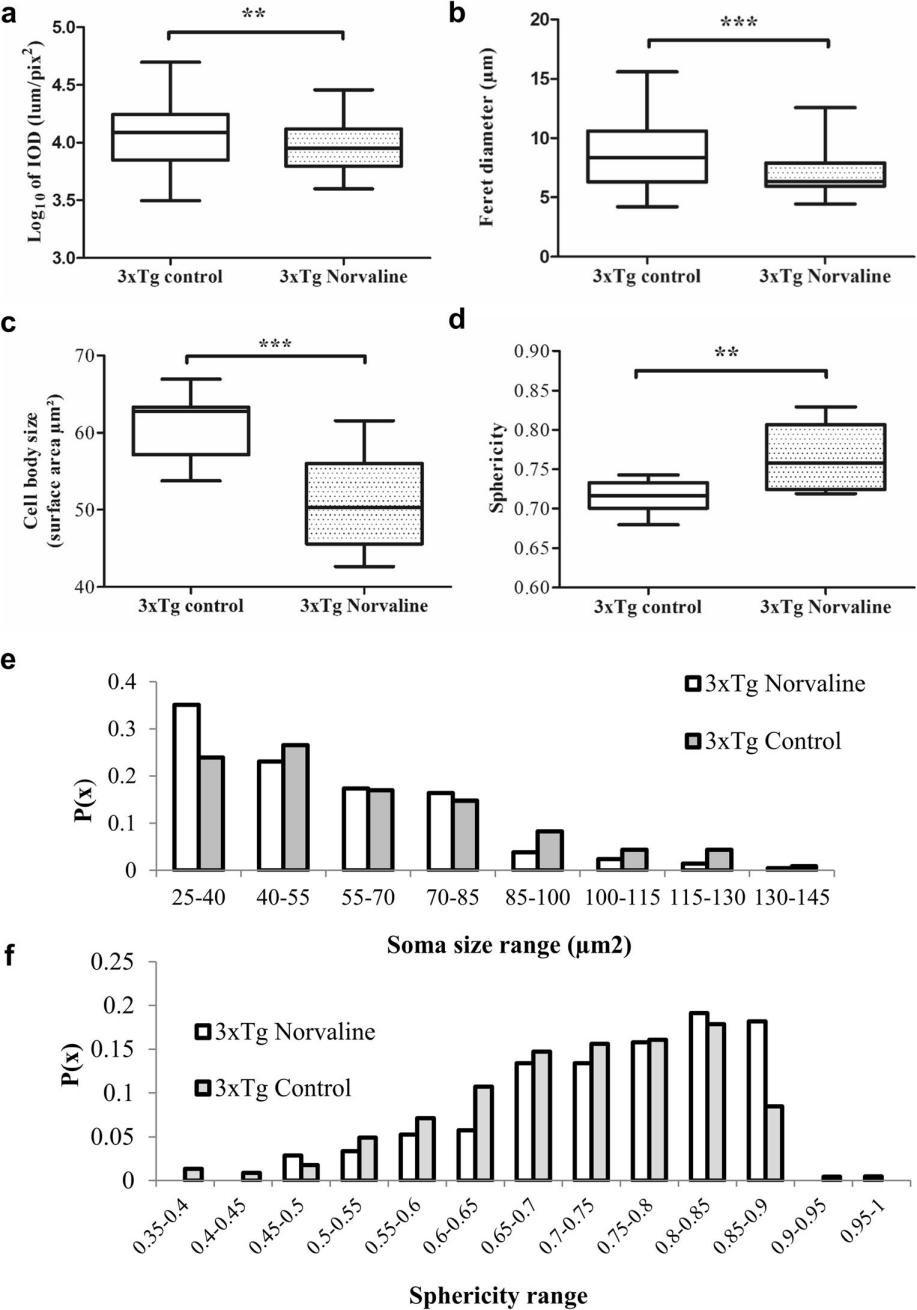
Quantitative characterization of microglial morphology in the CA3 hippocampal area with *S* = 0.2 mm^2^. Five sections per mice were included in the analysis. **(a)** IOD, **(b)** Feret’s diameter, **(c)** cell surface area, and **(d)** sphericity index. A frequency histogram of all microglial cells sampled in the CA3 area with *S* = 0.2 mm^2^. Data are presented via box and whisker graphs. The boxes extend from the 25th to 75th percentiles. The line in the middle of the box is plotted at the median. The whiskers denote the smallest and largest values. **(e)** The frequency distribution analysis of the soma area showed a shift from large to smaller cell body sizes after the treatment, and **(f)** the distribution analysis of circularity indicated that control cells were more likely to possess a more irregular shape (i.e., to have a lower sphericity index)

Microglial morphology and function are closely related. Microglia are sensitive to the surrounding microenvironment, and various stimuli regulate their morphology [56]. Neuroinflammation is characterized not only by an increase in microglial density but by diverse morphological changes, including de-ramification [57]. To address this issue, several morphological features of stained microglia were analyzed using image analysis software Image-Pro® 10 (Media Cybernetics, Rockville, USA) and ZEN 2.5 (Zeiss, Oberkochen, Germany). Iba1 is known to be expressed weakly by ramified microglia but upregulated and strongly expressed by activated microglia [58]. We used the IOD index to characterize Iba1 staining intensity [59]. The results revealed a significant treatment-related effect on the microglial IOD (Fig. 7a). In the treated group, this parameter was reduced by 30%.

A detailed analysis of microglial somata revealed several other treatment-related effects. First, the average size (surface area) of the measured cells in the treated group contracted by 17% (50.92 ± 1.69 μm^2^ in the treatment group *vs* 60.93 ± 1.06 μm^2^ in the untreated group; *p* < 0.0001) (Fig. 7c). Second, the roundness or sphericity of the somata (4π area/perimeter^2^), which reflects microglial reactivity, increased significantly in the L-norvaline-treated group (0.763 ± 0.012 in the treatment group *vs* 0.71 ± 0.005 in the untreated group; *p <* 0.0037) (Fig. 7d). Additionally, we measured the Feret diameter or max–min caliper. The analysis revealed a significant reduction in Feret’s diameter in the treated group (6.82 ± 0.182 μm *vs* 8.61 ± 0.26 μm; *p* < 0.0001) (Fig. 7b).

Furthermore, the distribution analyses revealed a left-shifted distribution of soma size and right-shifted distribution of circularity in the treated group (Fig. 7e, f). Together, these findings suggested that the number of microglia with a small, round cell body (resting microglia) increased after the treatment. The cells in the control group had a bigger and more irregular soma shape (activated microglia) as compared with those in the treated group. The observations herein were in accord with the common classification of microglia morphology, in which resting cells are considered small, round cells with elaborate ramifications, and activated cells are considered amoeboid, with retracted processes [60].

### L-Norvaline Reversed Astrocyte Degeneration in Brain Areas of the 3×Tg Mice, with Pronounced β-Amyloidosis

**Fig. 8:**
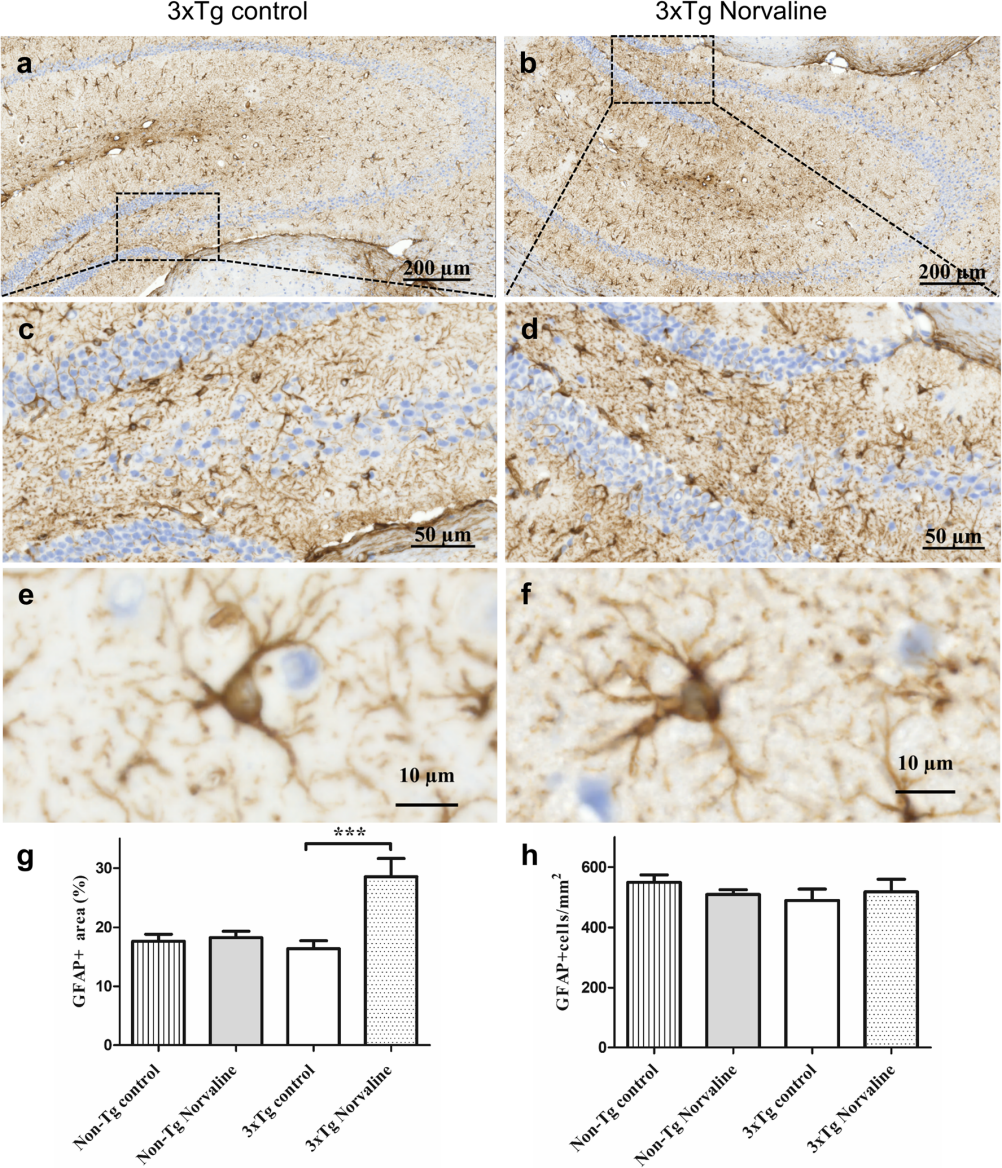
The L-norvaline did not lead to changes in GFAP-positive astroglial density in the 3×Tg mice but led to a significant increase in the volume of somata of astrocytes. Representative hippocampal bright-field × 20 micrographs of the hippocampi from the 3×Tg control **(a)** and L-norvaline-treated mice **(b)**. The hilus area of the control **(c)** and treated **(d)** 3×Tg mice. Representative × 40 images of astrocytes in the CA4 area of the control **(e)** and treated **(f)** mice. **(g)** Bar charts show a significant increase in the area fraction (%) of GFAP-positive cells in the hippocampal hilus area of the 3×Tg mice treated with L-norvaline. **(h)** GFAP-positive cell density in the hilus. *n* = 20, 4 mice per group. ***p < 0.001

Astrocytes modulate and control synaptic activity [61]. GFAP is highly expressed in astrocytes [62]. We stained the brains and observed GFAP immunoreactive cells with a typical stellate shape (Fig. 8e, f). Of note, the shape of the astrocytes in brain sections from the 3×Tg mice differed from those in non-Tg animals, with the astrocytes of the non-TG mice displaying a highly ramified star-shaped configuration (Supplementary Fig. S4). The astroglia in brain sections from the control 3×Tg mice showed fewer processes and a reduced volume (Fig. 8a, c) as compared with astroglia in brain sections from the animals treated with L-norvaline (Fig. 8b, d). We quantified the levels of GFAP immunoreactivity (reflected by the volume density) within the hilus area. The results of a comparative analysis using a one-way ANOVA (*F*(3,51) = 9.263, *p* < 0.0001), followed by Tukey’s multiple comparison tests between the experimental groups, revealed significantly increased (*p* < 0.001) glial somatic volumes in the 3×Tg animals treated with L-norvaline (Fig. 8g). The analysis did not reveal any significant changes in the number and surface area of GFAP-positive objects in the non-Tg mice following the treatment.

In addition, we measured the astrocyte density within the hilus. According to the literature, the average surface area of an astrocyte is about 250 μm^2^ [63]. To count the cells, we filtered out all GFAP-positive objects less than 70 and more than 500 μm^2^. The results revealed no significant differences (*p* = 0.55) in the density of GFAP-positive astrocytes between the control and experimental groups (Fig. 8h).

### ARGI Was Distinctly Expressed in Areas with Pronounced Amyloidosis, and the L-Norvaline Treatment Effectively Reduced ARGI Expression

**Fig. 9:**
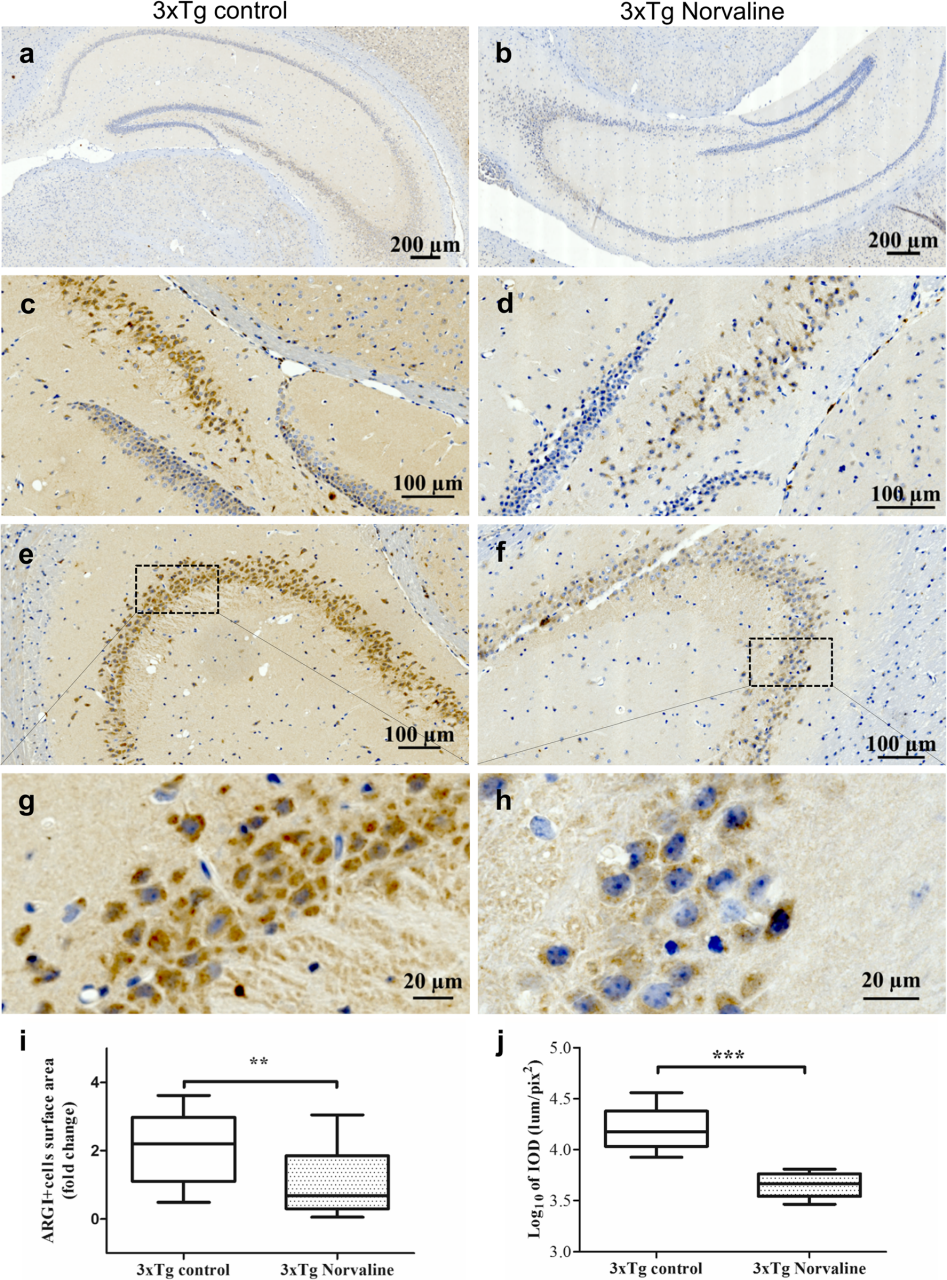
L-Norvaline significantly decreased ARGI immunopositivity in the 3×Tg mice. Representative hippocampal bright-field × 20 micrographs of the control **(a)** and L-norvaline-treated 3×Tg mice **(b).** CA4 area of the control **(c)** and treated **(d)** 3×Tg mice. CA3 area of the control **(e)** and treated **(f)** mice, with × 40 insets **(g)** and **(h),** respectively. **(i)** The boxplots show a significant reduction in the ARGI-positive cell surface area in the CA3 areas (*S* = 0.2 mm^2^) and **(j)** the IOD of ARGI-positive cells in the CA3 area in the 3×Tg mice treated with L-norvaline. Student’s *t* test (*n* = 24, with 4 mice per group), **p < 0.01, ***p < 0.001

We examined ARGI protein spatial expression within the hippocampi of the 3×Tg mice and its spatial relationship with Aβ deposition using immunocytochemistry (Fig. 9a, b). ARGI preferentially accumulated in the CA1–CA4 areas of the hippocampus (Fig. 9c–f) and was mainly detected inside the cells (Fig. 9g, h). The L-norvaline treatment led to a significant (*p* = 0.0073) reduction (more than 2-fold) in the overall ARGI-positive cell surface area (Fig. 9i) and IOD (*p* = 0.0004) (Fig. 9j), which reflected the decrease in the levels of the ARGI protein in the brains of the treated animals.

In the non-Tg mice, hippocampal pyramidal neurons expressed a significantly lower level of the ARGI protein as compared with that in hippocampal pyramidal neurons of the 3×Tg mice (Supplementary Fig. S5). No differences in the level of immunopositivity were detected in the treated and control groups using the same software and the same threshold. Of note, several ARGI-positive star-shaped cells with dark nuclei were seen in the hippocampus (Fig. S5 insets).

### Mitochondrial ARGII Was Expressed in the Cytosol of CA2–CA3 Hippocampal Cells

**Fig. 10:**
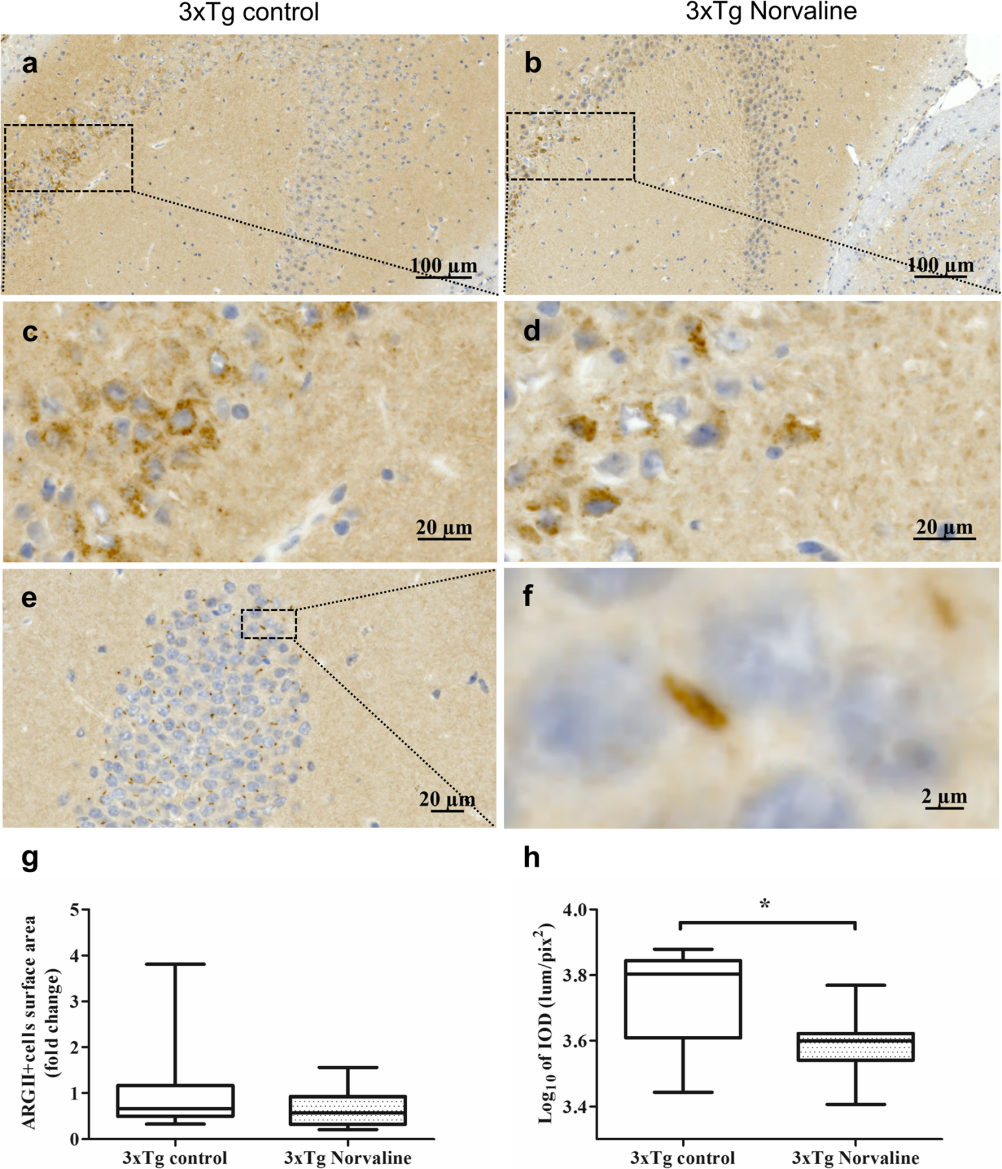
Representative hippocampal CA2–CA3 bright-field × 20 micrographs of the control **(a)** and L-norvaline-treated 3×Tg mice **(b)**. CA2 × 40 insets of the control **(c)** and treated **(d)** mice. **(e)** A representative × 20 image of the dentate gyrus, with mitochondria stained with ARGII antibody **(f)** and a × 40 zoomed-in inset clearly showing a mitochondrion. **(g)** ARGII-positive cell surface area in the CA2 (*S* = 0.2 mm^2^). **(h)** The IOD of ARGII-positive cells in the CA2 area. Student’s *t* test, *p < 0.05, *n* = 12, with 4 mice per group

We detected augmented ARGII immunopositivity of cells in the CA2 hippocampal area of the 3×Tg mice (Fig. 10a–d) but not in that of the non-Tg animals (Supplementary Fig. S6). The same pattern of staining was observed in the treated and control groups. At × 40 and higher magnification, mitochondria were observed, most clearly in the dentate gyrus, without significant differences in the numbers and intensity between the groups (Fig. 10e, f). We did not detect any significant effect of the treatment on the “ARGII-positive objects surface area” (Fig. 10g). However, the IOD index was significantly reduced (*p* < 0.05) in the L-norvaline-treated 3×Tg mice (Fig. 10h).

## Discussion

In this study, we treated 3×Tg mice with a nonproteinogenic unbranched-chain amino acid L-norvaline, which possesses arginase-inhibiting properties [64]. The results indicated that L-norvaline was well tolerated by the mice and that long-term treatment with L-norvaline did not lead to detectable behavioral changes or weight loss in WT mice. The 3×Tg mice in the L-norvaline-treated group not only exhibited improved spatial learning and memory but also a reduction in the cerebral Aβ burden, together with a substantial decrease of Aβ toxic fibrillary and oligomeric species. Moreover, microglial density was decreased in the treated 3×Tg mice, and the activated microglial phenotype shifted to a resting phenotype.

Blocking L-arginine depletion and reversal of its deprivation, thereby halting memory loss and reducing other AD symptoms, has been explored previously. For example, in a murine model of AD, Kan et al. [17] used DFMO, a relatively toxic, mostly irreversible, inhibitor of ornithine decarboxylase and putrescine in AD. In the present study, we used L-norvaline to inhibit arginase. L-Norvaline is structurally similar to ornithine and acts via a negative feedback inhibition mechanism. Accordingly, we did not administer polyamines to avoid the undesirable side effects of DFMO. Furthermore, we dissolved L-norvaline in water, and the animals received the agent via water in their home cages. This mode of administration is much less invasive than forced gavage feeding. Furthermore, we utilized a universally recognized animal model of AD.

The mice in the L-norvaline-treated group showed a very strong phenotype in 2 different behavioral paradigms, which supports the effect of the treatment on short- and long-term spatial memory acquisition. Moreover, we found a significant reduction in the level of 6E10 positivity in the cortices of the 3×Tg mice treated with L-norvaline. Also, we found evidence that the hippocampi of the 3×Tg-treated mice at age 6–7 months harbored significantly lower levels of Aβ oligomeric and fibrillary conformers as compared with hippocampi of the untreated animals. A previous study demonstrated that soluble low-weight Aβ oligomers and intermediate aggregates were the most neurotoxic forms of Aβ and that these triggered synaptic dysfunction and neuronal damage, which manifested in behavioral attributes of AD, such as memory deficits [65].

Furthermore, in the present study, we detected a substantial increase in dendritic spine density in the hippocampi of the 3×Tg mice treated with L-norvaline. In addition, we evidenced a significant rise in the levels of pre- and postsynaptic proteins in the experimental group. We attributed this rise to an increase in spine density.

In this study, ARGI expression was significantly increased in areas with pronounced Aβ deposition. There is a consensus in the literature that various stimuli can induce the expression of the 2 isoforms of arginase [66]. In an AD murine model, ARGI was localized not only in brain cells but also distributed in extracellular spaces [17]. In the hippocampus, ARGI displayed a spatial correlation with Aβ deposition and Iba1 expression [17]. The activation of ARGII was shown to be associated with translocation from the mitochondria to the cytosol [22].

The immunohistochemistry approaches applied herein revealed significantly increased ARGI protein levels in the CA2–CA4 hippocampal areas of the 3×Tg mice, where we detected the most distinct intracellular Aβ deposition. These findings are in accordance with those in the literature [17]. Of note, in the present study, ARGI staining was primarily localized inside the cells. The discord between these findings and those of the earlier study may be attributed to differences in the murine models of AD. Furthermore, the L-norvaline treatment significantly reduced the level of ARGI expression in the hippocampus.

Additionally, we observed augmented ARGII immunopositivity in cells in the CA2 area of the 3×Tg mice. This finding pointed to the translocation of ARGII from the mitochondria to the cytoplasm, presumably by the same mechanism described previously [22]. However, we did not detect between-group differences in the relative surface area of ARGII-positive objects located in the CA2 area, although the optical density of the objects was significantly reduced in the treated mice.

Chronic neuroinflammation is a prominent feature of AD pathogenesis [67]. Microglial proliferation is increased in AD patients, as well as in AD animal models, and it is positively correlated with disease severity [68]. It has been hypothesized that activated microglia cause synaptic and wiring dysfunction by pruning synaptic connections [69] and that targeting the microglia–synapse pathways might prevent AD symptoms [70]. In the present study, we observed an increase of more than 30% in the density of Iba1 immunopositive cells in the CA3 area of the hippocampi of 7-month-old 3×Tg mice as compared with that in age- and sex-matched non-Tg controls. This finding is in accordance with the literature [71]. Moreover, we confirmed that cognitive function improvements in the mice treated with L-norvaline were associated with a significant reduction in hippocampal microgliosis.

In parallel with microgliosis and astrogliosis [72], the development of AD is associated with astrodegeneration [73]. Astrocytes are essential for neurotransmission, and astrodegeneration leads to synaptic strength deficits and contributes to the decline in the number of active synapses observed in the early stages of AD [74]. Atrophy of astrocytes was previously detected in the hippocampi and cortices of 3×Tg mice [75]. Astrocytic atrophy was characterized by a reduction in cell volume and a decrease in the number of processes [76]. Interestingly, the density of astrocytes remained relatively stable in the hippocampus and cortices of 3×Tg mice for more than 1 year and was comparable to that observed in non-Tg controls [77]. Olabarria et al. [78] reported an apparent reduction in the astrocytic somatic volume in the dentate gyrus of 6-month-old 3×Tg mice and a significant decrease at 12 months. The results of our study, which revealed astrodegeneration in areas with intense Aβ deposition, are in accordance with the data presented in the literature. Moreover, the L-norvaline treatment led to a significant (75%) increase in the GFAP-positive surface area in the hilus.

L-Norvaline possesses multiple mechanisms of activity. Ming et al. [33] demonstrated that L-norvaline inhibited endothelial inflammation induced by tumor necrosis factor-α independently of NOS and arginase activity. The authors utilized RNA interference technology to prove that the anti-inflammatory properties of L-norvaline were attributed to its ability to inhibit the S6K1 kinase, which is involved in the regulation of protein synthesis. Subsequently, Caccamo et al. provided evidence that the activity of S6K1 was higher in 3×Tg mice as compared with that in WT mice [79]. The authors also showed that removing 1 copy of the S6K1 gene from the 3×Tg mice was sufficient to rescue long-term potentiation (LTP) deficits. They speculated that the improvement in LTP corresponded to observed changes in the expression of the synaptic marker synaptophysin, which was reduced in 3×Tg mice but increased dramatically in S6K1-deficient mice. In the present study, the levels of synaptophysin in the hippocampi of the 3×Tg mice treated with L-norvaline increased by 50%. This finding agrees with the data presented in the aforementioned literature.

The observation of a significant (*p* = 0.038) reduction by 53% in RAC-α protein-serine/threonine kinase (Akt1) levels in the treated group is noteworthy (Supplementary Table 2). This kinase is a key modulator of the AKT–mTOR signaling pathway and regulates many processes, including metabolism, proliferation, and cell survival [80]. As it is located upstream of mTOR and S6K1, downregulation of Akt1 can negatively influence S6K1 activity. Upregulation of mTOR signaling pathway is known to play a central role in major pathological processes of AD [81]. Consequently, inhibition of mTOR is a novel therapeutic target for AD [82].

Overall, our results provide compelling evidence that L-norvaline is a multifunctional agent and a potential drug for the treatment of AD. It appears to be a promising candidate for clinical development. The functional effects of L-norvaline are diverse, and much work remains to be done to disclose its full potential and precise mechanisms of action.

## Electronic Supplementary Material

Fig. S1 Representative hippocampal 20× bright-field micrographs of immunohistochemical staining of brain slices from 3×Tg controls for Iba1 **(a)**, GFAP **(c)**, ARGI **(e)**, and ARGII **(g),** with corresponding negative controls **(b)**, **(d)**, **(f)**, **and (h)**. As a negative control, serial sections were processed without primary antibody. (PNG 19829 kb)

High Resolution Image (TIF 23938 kb)

Fig. S2 Western blots of A11 **(a)**, OC **(b)**, and β-actin. (PNG 3545 kb)

High Resolution Image (TIF 6087 kb)

Fig. S3 KCPS Western blots. **(a)** Control. **(b)** Treatment. Lane 2: nerve growth factor receptor-tyrosine kinase, lane 3: cyclin E, lane 4: protein-serine phosphatase 2A - B regulatory subunit - B56 alpha isoform, lane 5: synapsin 1 isoform 1a, lane 6: neuroligin-1, lane 7: vesicular glutamate transporter 3, lane 8: synaptophysin, lane 9: vesicular glutamate transporter 1, lane 10: short transient receptor potential, lane 11: snaptotagmin-10, lane 12: proto-oncogene tyrosine-protein, lane 13: Synaptotagmin-6, lane 14: voltage-dependent L-type calcium channel, lane 15: synaptotagmin-12, and lane16: β-actin. (PNG 769 kb)

High Resolution Image (TIF 1771 kb)

Fig. S4 Representative immunolabeling with the astrocyte marker GFAP in bright-field 40× micrographs of the hippocampus from non-Tg control mice **(a)**. A typical star-shaped astrocyte in the CA1 region is shown in the inset. A zoomed-in micrograph of the hilus **(b)** with a typical astrocyte **(c)**. (PNG 14955 kb)

High Resolution Image (TIF 19106 kb)

Fig. S5 Representative ARGI-labeled hippocampal bright-field 20× micrograph from non-Tg control mice. Hippocampal pyramidal neurons expressed significantly less ARGI protein as compared with those in the 3×Tg mice (Fig. 9). Using the same software, no differences were detected in the level of immunopositivity of the treated and control groups (not shown). However, several ARGI-positive star-shaped cells possessing dark nuclei were observed in the CA2 area and in the proximity of the CA3 area (40× insets). (PNG 8644 kb)

High Resolution Image (TIF 12525 kb)

Fig. S6 Representative ARGII-labeled bright-field 20× micrograph of the hippocampus from non-Tg control mice. No ARGII protein was detected in the cytoplasm of hippocampal pyramidal neurons. However, ARGII-positive mitochondria were clearly seen in the dentate gyrus and CA3 area (40× insets). (PNG 12615 kb)

High Resolution Image (TIF 15842 kb)

Table S1 KCPS primary antibody selection. (DOCX 14 kb)

Table S2 Selected results of the antibody array. Only proteins with a significant (*p* < 0.05) fold change from the control (CFC) are displayed (except for neuroligin, which exhibited 252% of change). The cut-off was set at ±19% change. (DOCX 19 kb)

ESM 1 (PDF 495 kb)
