# Supplementary figures and images for: L-Norvaline Reverses Cognitive Decline and Synaptic Loss in a Murine Model of Alzheimer’s Disease
Source: Neurotherapeutics. 2018 Oct 4;15(4):1036–54. doi: 10.1007/s13311-018-0669-5 (PMC6277292; doi:10.1007/s13311-018-0669-5)

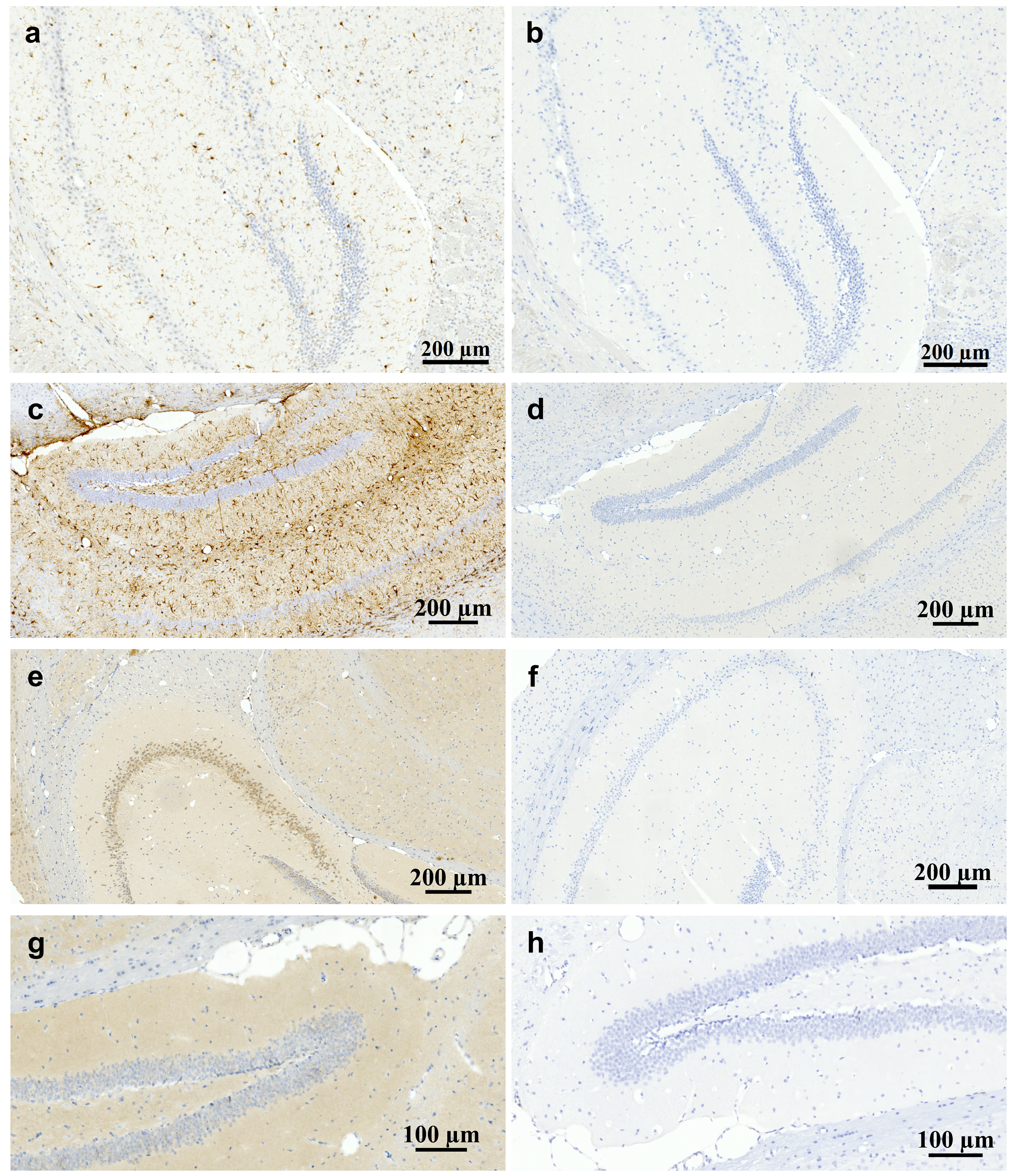

Supplement: Supplementary file 1 — Representative hippocampal 20× bright-field micrographs of immunohistochemical staining of brain slices from 3×Tg controls for Iba1 (a), GFAP (c), ARGI (e), and ARGII (g), with corresponding negative controls (b), (d), (f), and (h). As a negative control, serial sections were processed without primary antibody. (PNG 19829 kb) [file 13311_2018_669_Fig11_ESM.png]

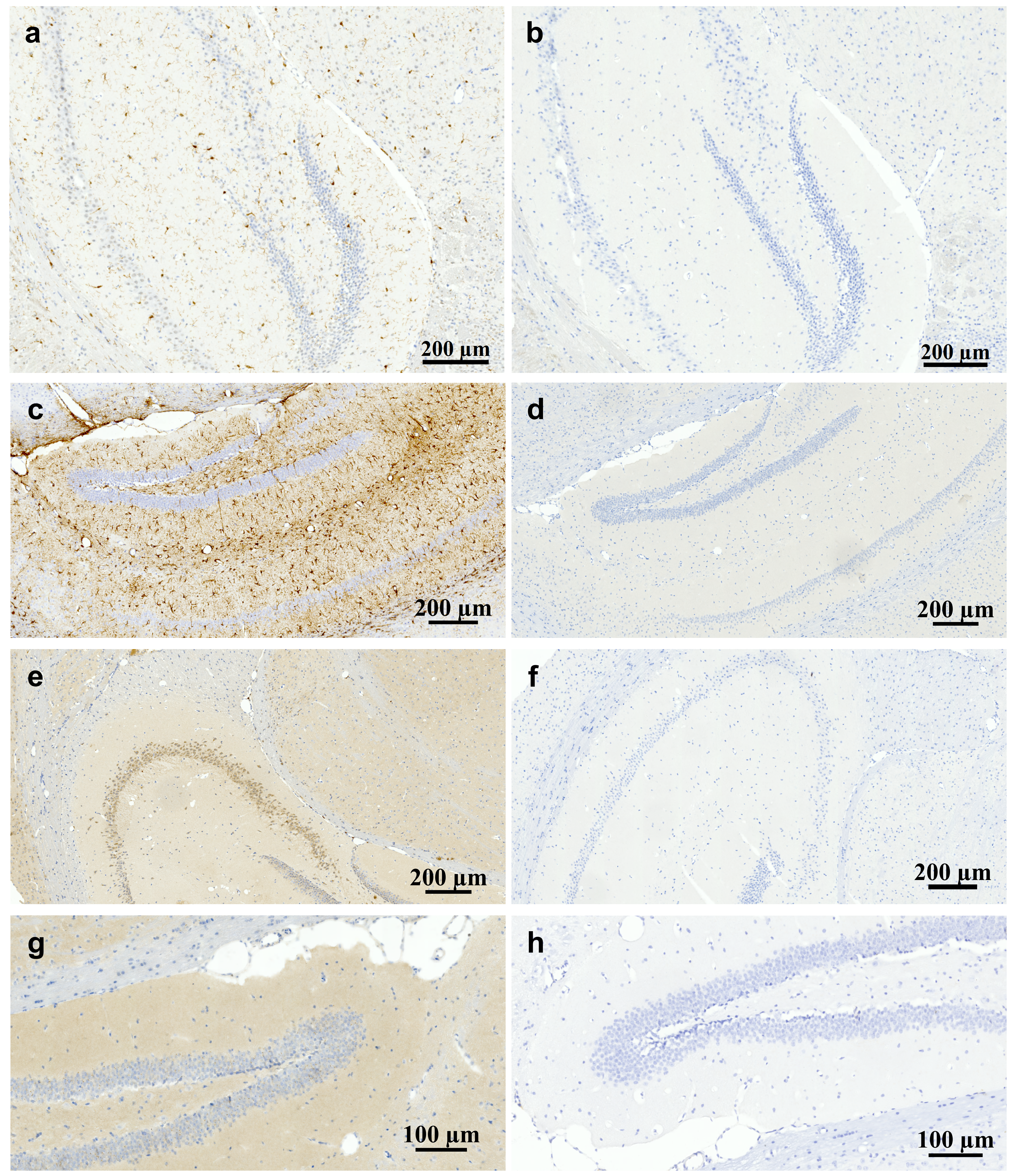

Supplement: Supplementary file 2 — High Resolution Image (TIF 23938 kb) [file 13311_2018_669_MOESM1_ESM.tif]

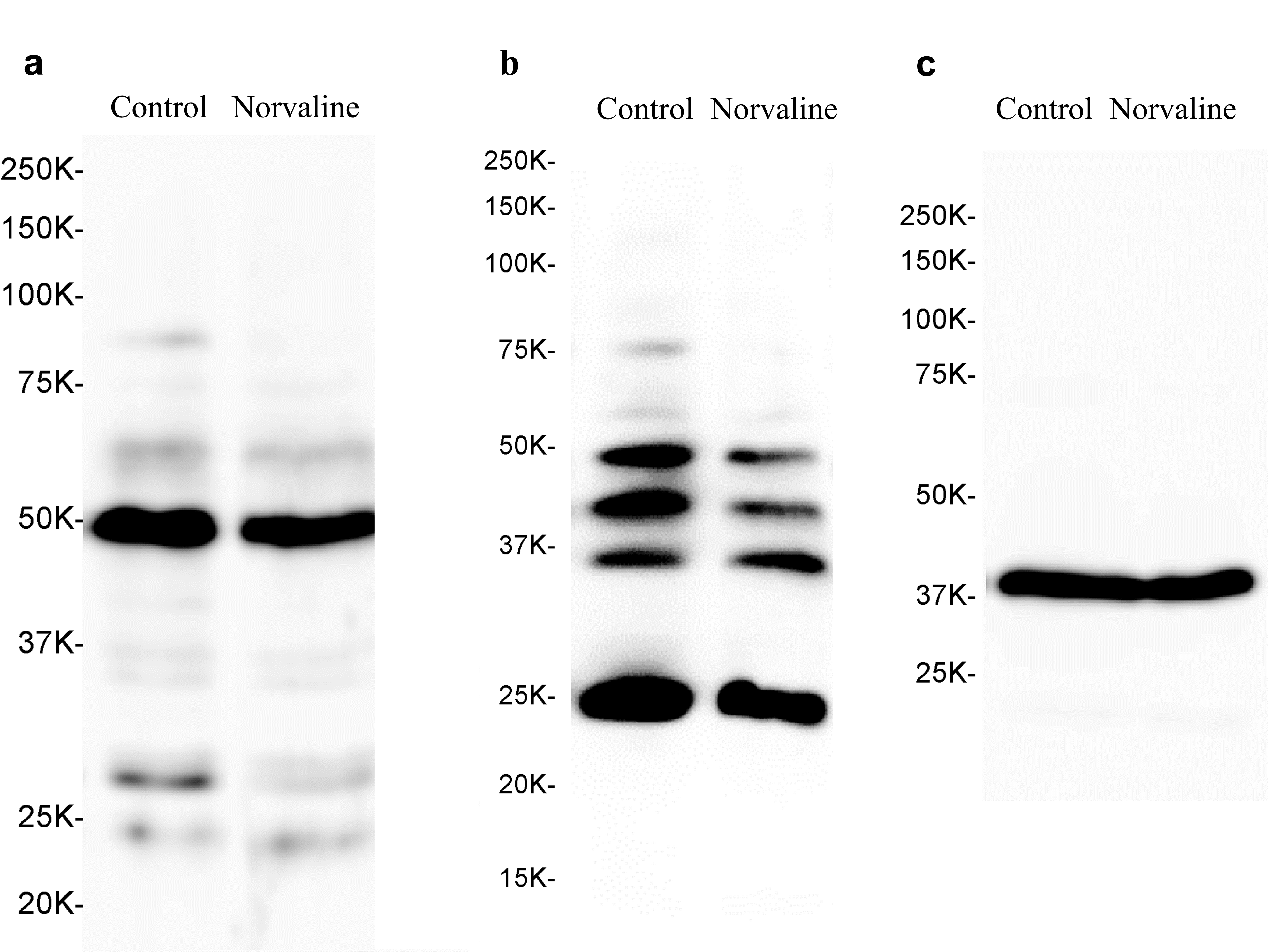

Supplement: Supplementary file 3 — Western blots of A11 (a), OC (b), and β-actin. (PNG 3545 kb) [file 13311_2018_669_Fig12_ESM.png]

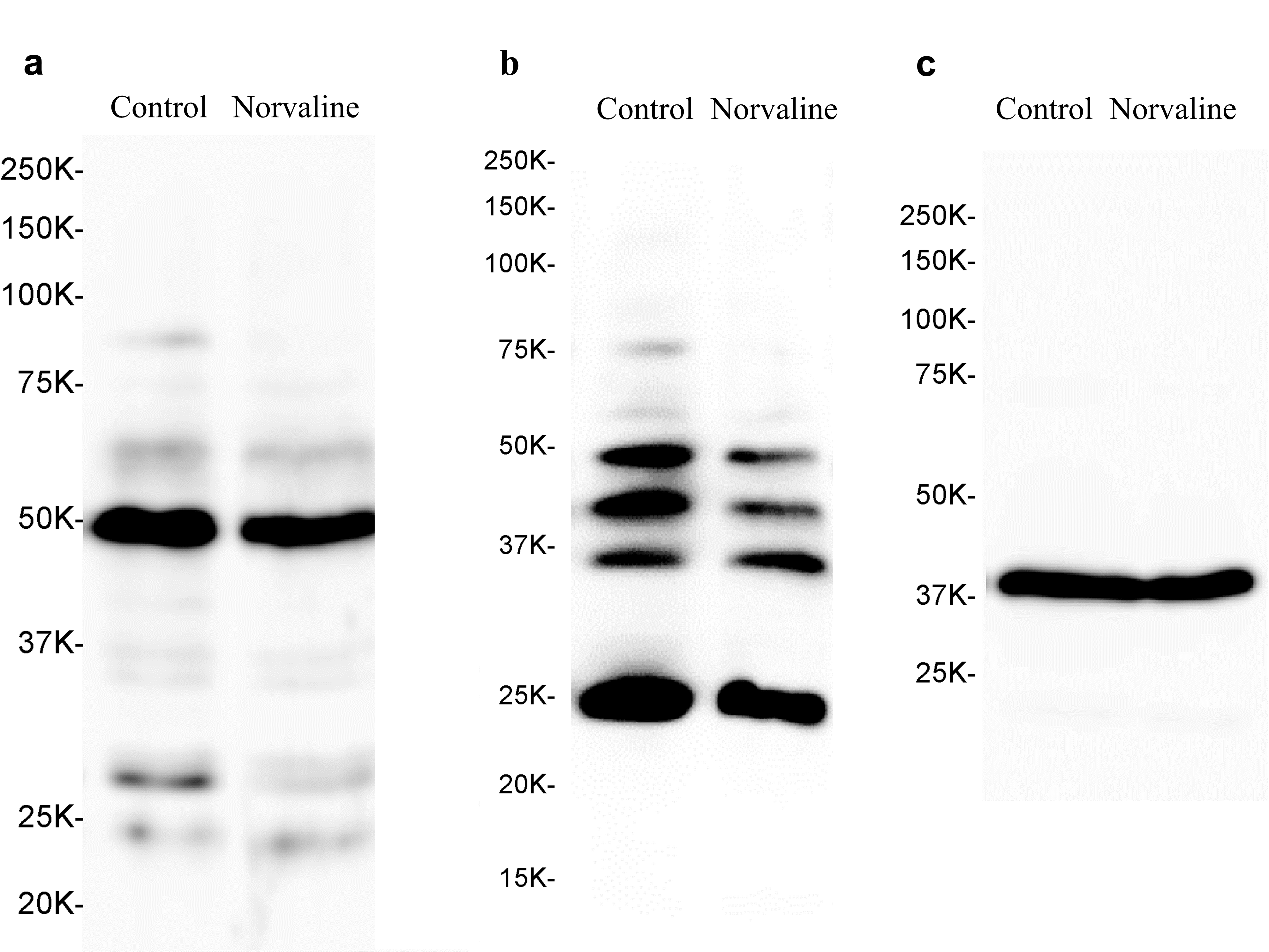

Supplement: Supplementary file 4 — High Resolution Image (TIF 6087 kb) [file 13311_2018_669_MOESM2_ESM.tif]

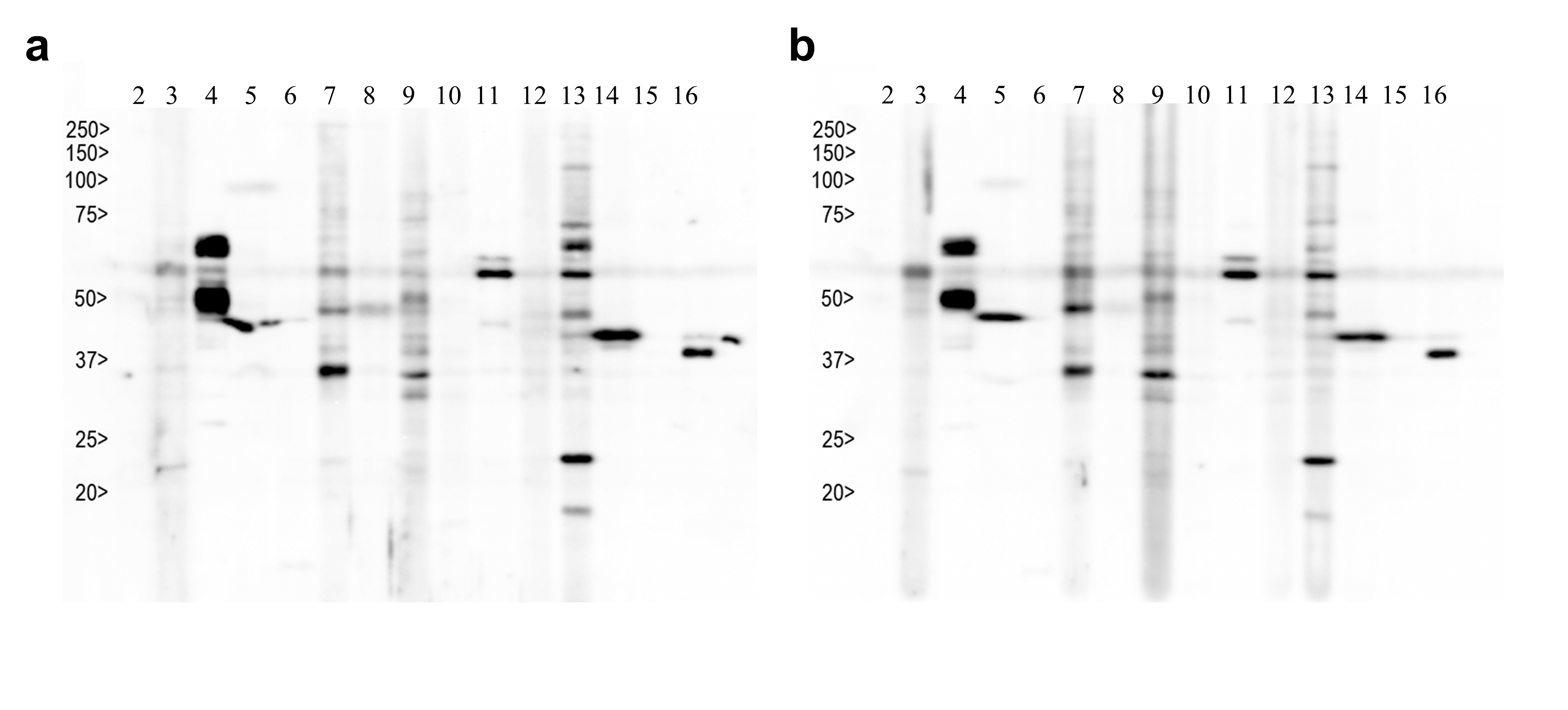

Supplement: Supplementary file 5 — KCPS Western blots. (a) Control. (b) Treatment. Lane 2: nerve growth factor receptor-tyrosine kinase, lane 3: cyclin E, lane 4: protein-serine phosphatase 2A - B regulatory subunit - B56 alpha isoform, lane 5: synapsin 1 isoform 1a, lane 6: neuroligin-1, lane 7: vesicular glutamate transporter 3, lane 8: synaptophysin, lane 9: vesicular glutamate transporter 1, lane 10: short transient receptor potential, lane 11: snaptotagmin-10, lane 12: proto-oncogene tyrosine-protein, lane 13: Synaptotagmin-6, lane 14: voltage-dependent L-type calcium channel, lane 15: synaptotagmin-12, and lane16: β-actin. (PNG 769 kb) [file 13311_2018_669_Fig13_ESM.png]

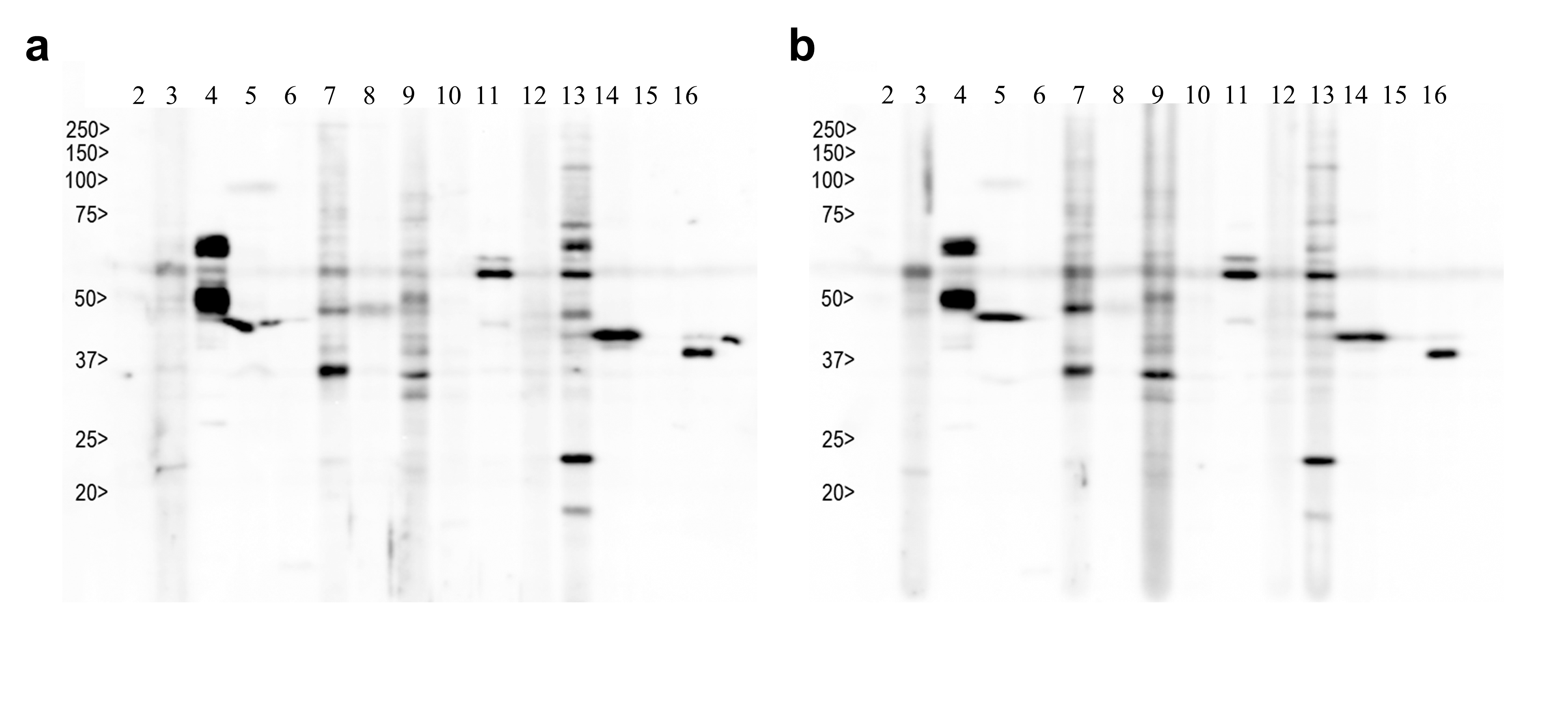

Supplement: Supplementary file 6 — High Resolution Image (TIF 1771 kb) [file 13311_2018_669_MOESM3_ESM.tif]

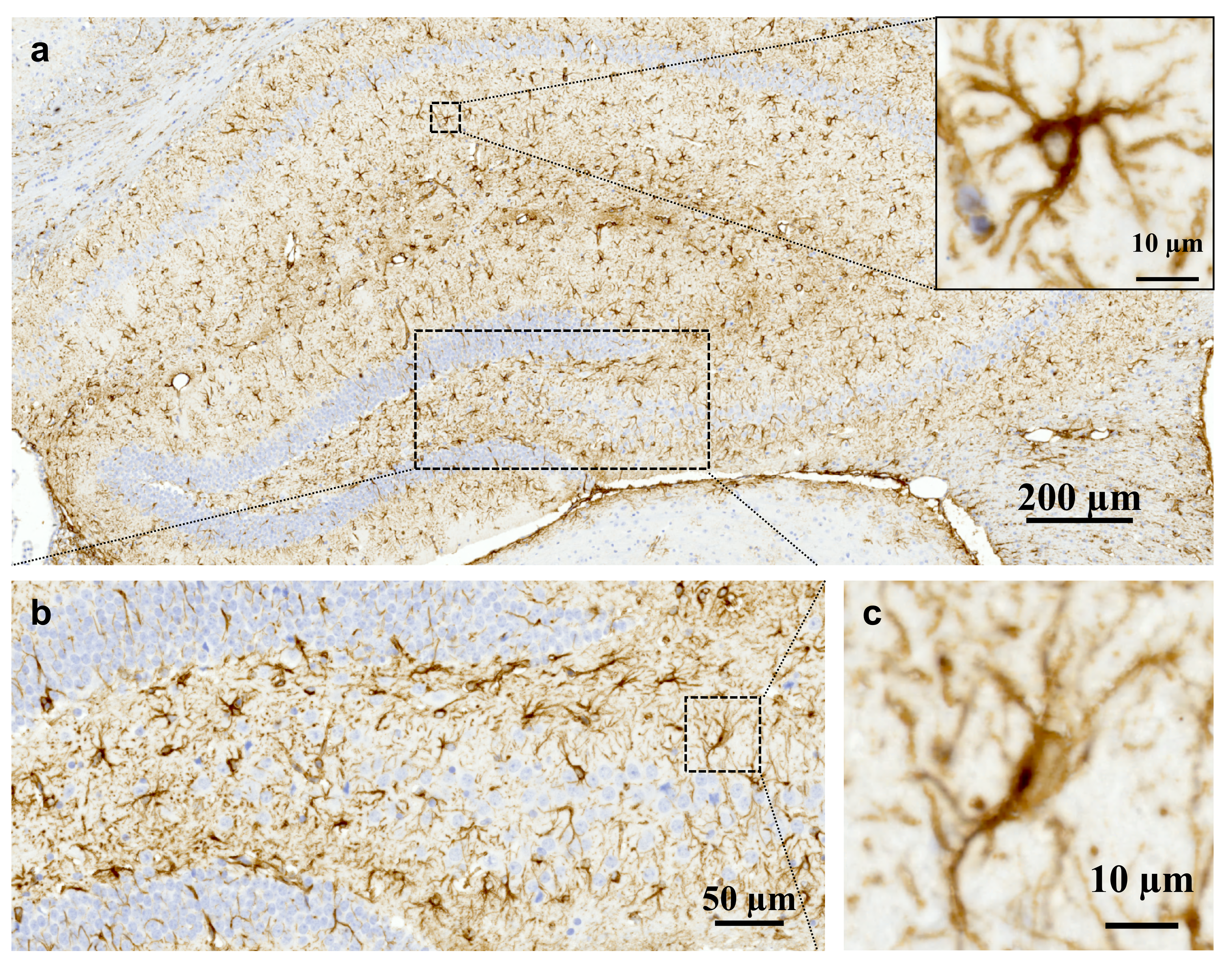

Supplement: Supplementary file 7 — Representative immunolabeling with the astrocyte marker GFAP in bright-field 40× micrographs of the hippocampus from non-Tg control mice (a). A typical star-shaped astrocyte in the CA1 region is shown in the inset. A zoomed-in micrograph of the hilus (b) with a typical astrocyte (c). (PNG 14955 kb) [file 13311_2018_669_Fig14_ESM.png]

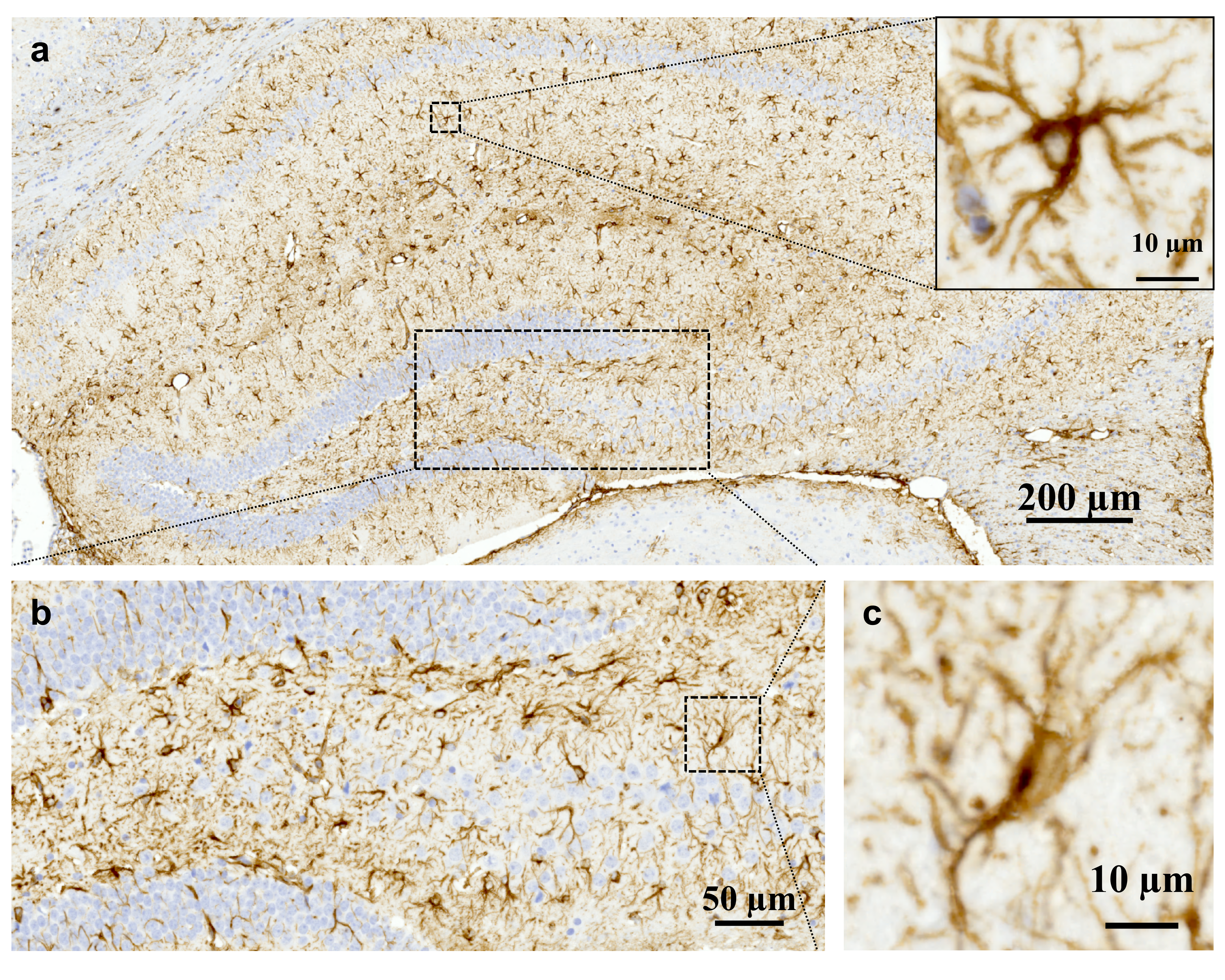

Supplement: Supplementary file 8 — High Resolution Image (TIF 19106 kb) [file 13311_2018_669_MOESM4_ESM.tif]

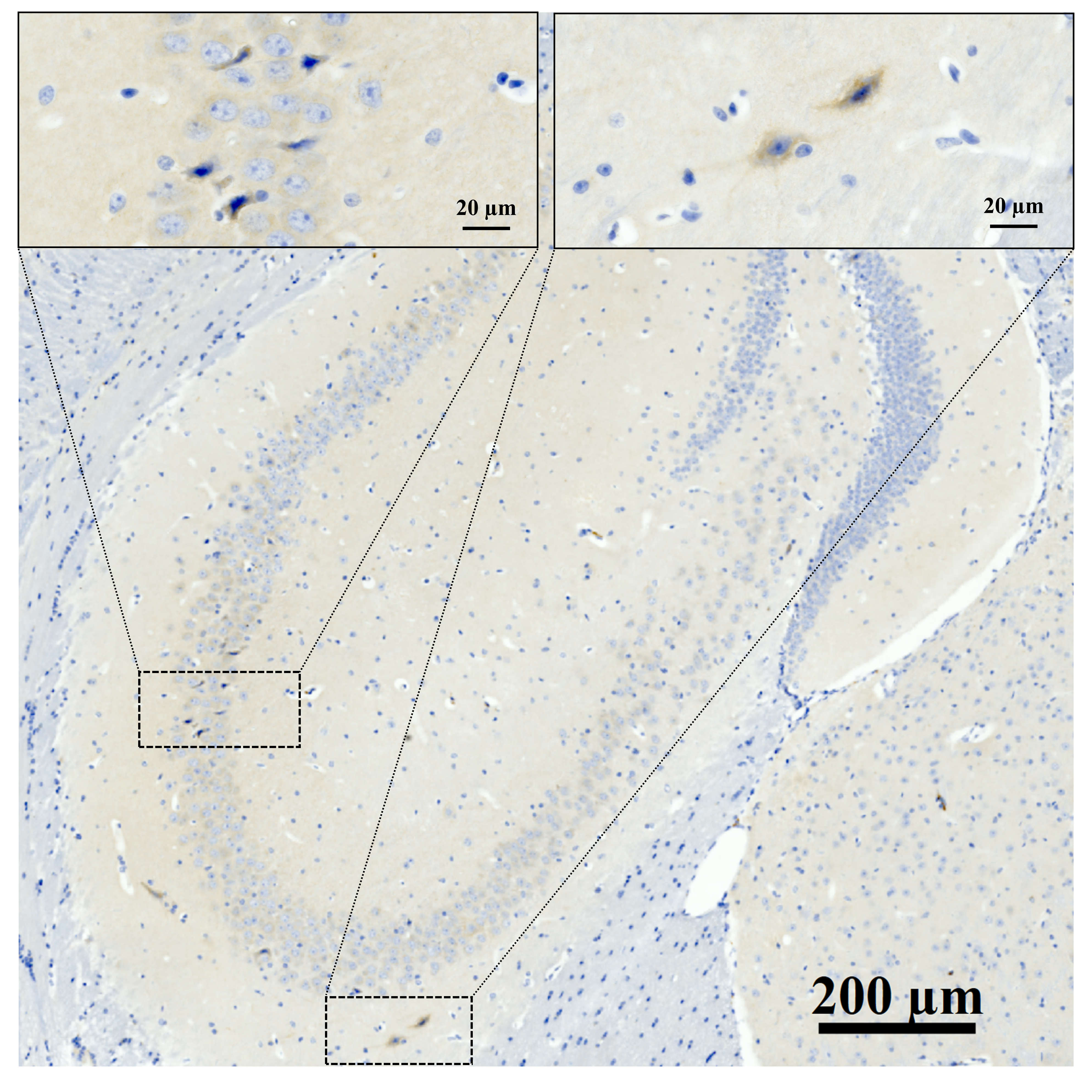

Supplement: Supplementary file 9 — Representative ARGI-labeled hippocampal bright-field 20× micrograph from non-Tg control mice. Hippocampal pyramidal neurons expressed significantly less ARGI protein as compared with those in the 3×Tg mice (Fig. 9). Using the same software, no differences were detected in the level of immunopositivity of the treated and control groups (not shown). However, several ARGI-positive star-shaped cells possessing dark nuclei were observed in the CA2 area and in the proximity of the CA3 area (40× insets). (PNG 8644 kb) [file 13311_2018_669_Fig15_ESM.png]

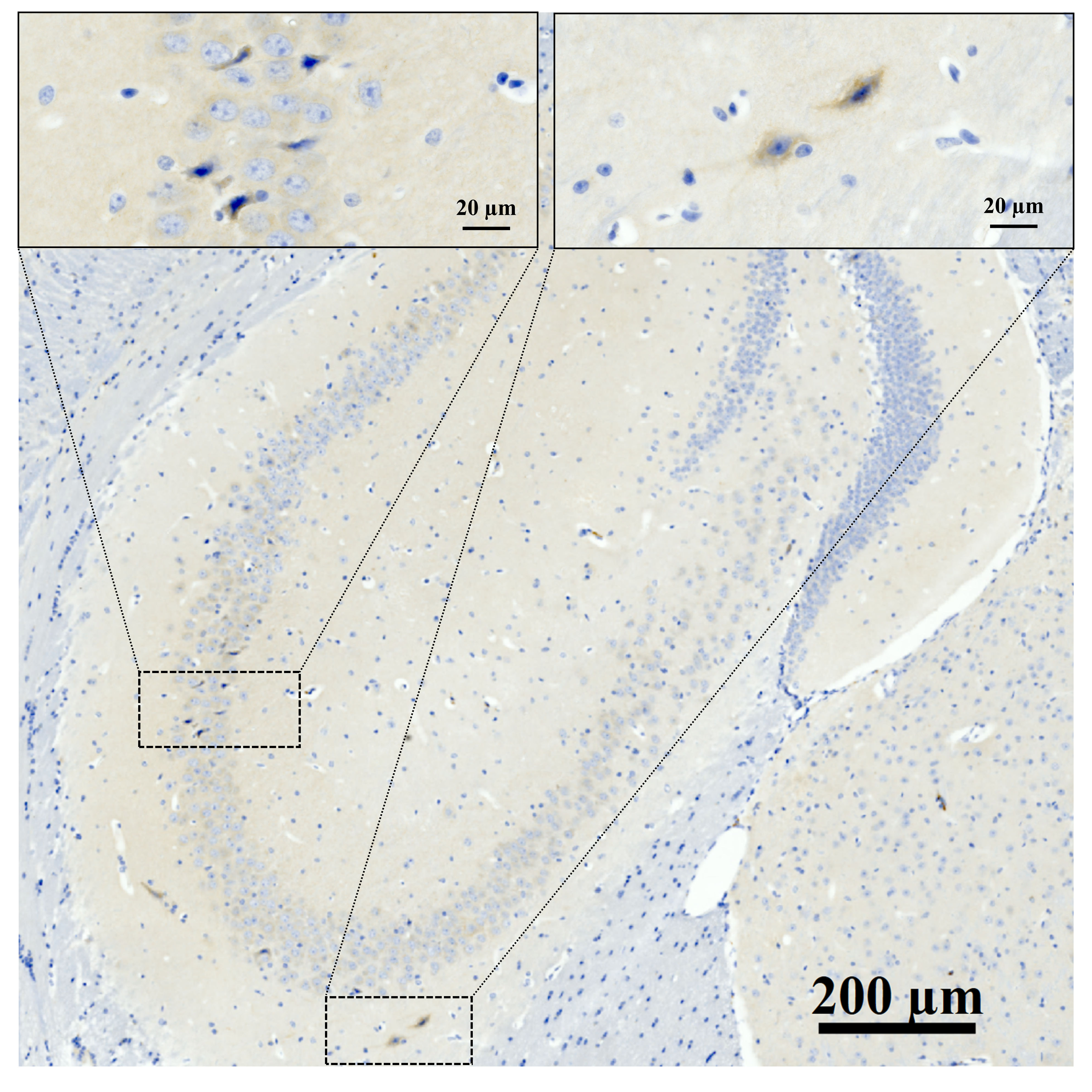

Supplement: Supplementary file 10 — High Resolution Image (TIF 12525 kb) [file 13311_2018_669_MOESM5_ESM.tif]

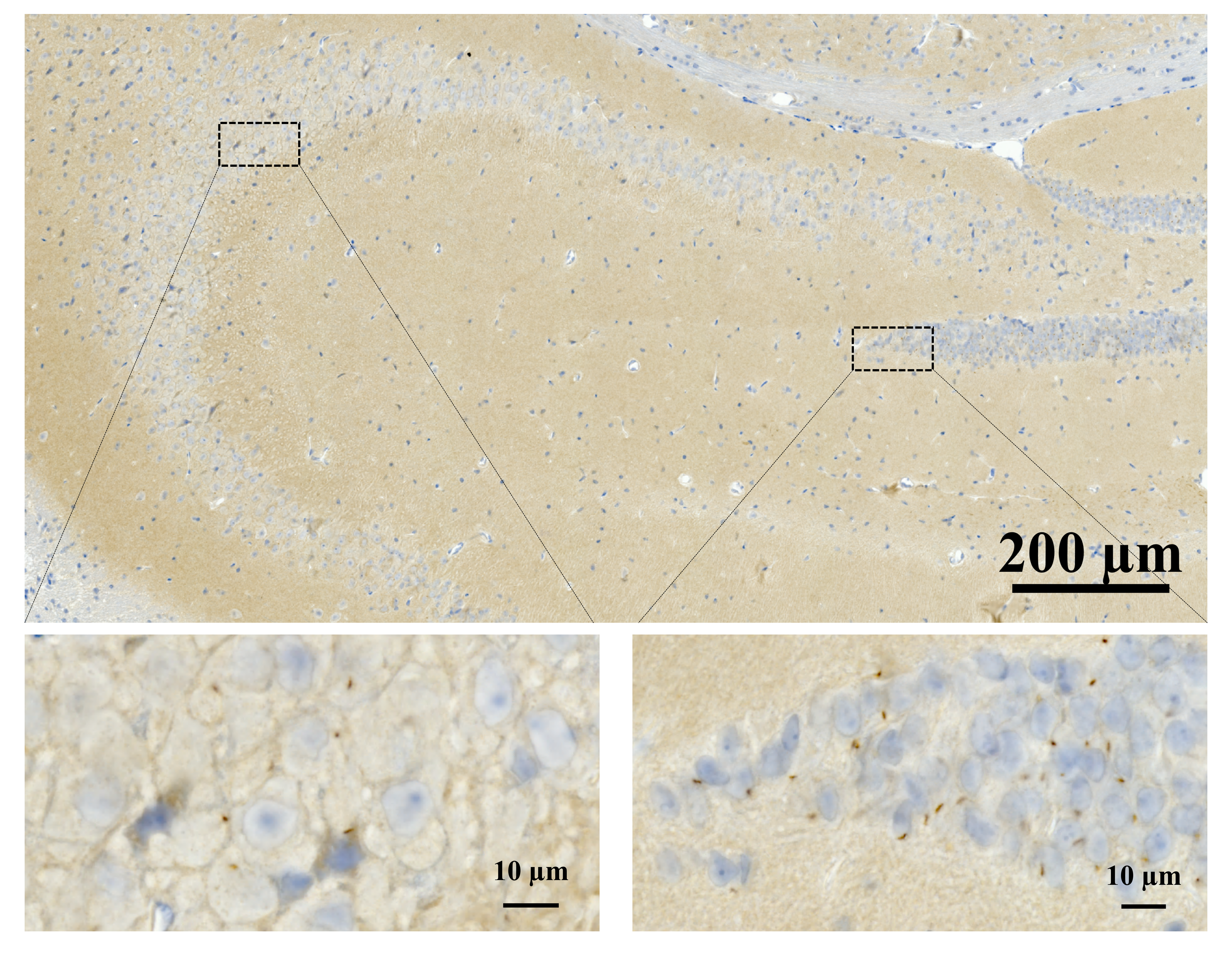

Supplement: Supplementary file 11 — Representative ARGII-labeled bright-field 20× micrograph of the hippocampus from non-Tg control mice. No ARGII protein was detected in the cytoplasm of hippocampal pyramidal neurons. However, ARGII-positive mitochondria were clearly seen in the dentate gyrus and CA3 area (40× insets). (PNG 12615 kb) [file 13311_2018_669_Fig16_ESM.png]

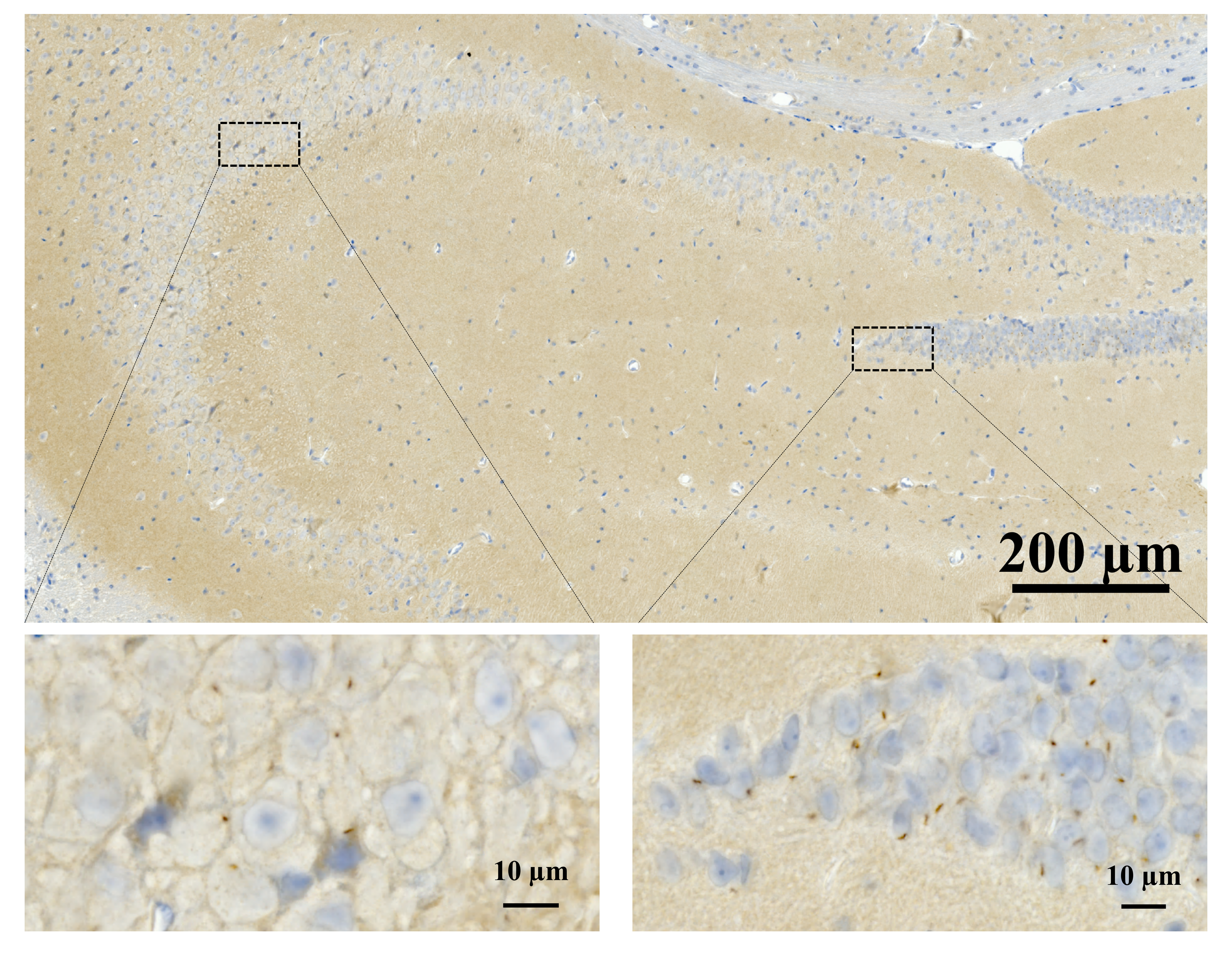

Supplement: Supplementary file 12 — High Resolution Image (TIF 15842 kb) [file 13311_2018_669_MOESM6_ESM.tif]
