# Supplementary material for: L-Norvaline Reverses Cognitive Decline and Synaptic Loss in a Murine Model of Alzheimer’s Disease
Source: Neurotherapeutics. 2018 Oct 4;15(4):1036–54. doi: 10.1007/s13311-018-0669-5 (PMC6277292; doi:10.1007/s13311-018-0669-5)
Supplement: Supplementary file 13 — KCPS primary antibody selection. (DOCX 14 kb) [file 13311_2018_669_MOESM7_ESM.docx]

| ***Ab Code*** | ***Type*** | ***Protein Name*** | ***Host Species*** | ***MW Range*** | ***Dilution*** | ***Ab Conc'n*** |
| --- | --- | --- | --- | --- | --- | --- |
| NK178 | pan-specific | TrkA | RpAb | 87 kDa | 1 : 250 | 4 µg/ml |
| NN031 | pan-specific | Cyclin E | MmAb | 47 kDa | 1 : 500 | 2 µg/ml |
| NP033 | pan-specific | PP2A B' (B56) | RpAb | 56 kDa | 1 : 3000 | unknown conc'n |
| NN171-2 | pan-specific | Synapsin 1 isoform 1a | MmAb | 74 kDa | 1 : 1000 | 1 µg/ml |
| NN294-1 | pan-specific | Neuroligin-1 | MmAb | 120 kDa | 1 : 250 | 4 µg/ml |
| NN362-1 | pan-specific | Vesicular glutamate transporter 3 | MmAb | 65 kDa | 1 : 250 | 4 µg/ml |
| N370-1 | pan-specific | Synaptophysin | MmAb | 38 kDa | 1 : 250 | 4 µg/ml |
| NN361-1 | pan-specific | Vesicular glutamate transporter 1 | MmAb | 60 kDa | 1 : 250 | 4 µg/ml |
| NN350-1 | pan-specific | Short transient receptor potential | MmAb | 110 kDa | 1 : 250 | 4 µg/ml |
| NN344-1 | pan-specific | Synaptotagmin-10 | MmAb | 60 kDa | 1 : 250 | 4 µg/ml |
| NN244-2 | pan-specific | Proto-oncogene tyrosine-protein | RpAb | 140 kDa | 1 : 250 | 4 µg/ml |
| NN367-1 | pan-specific | Synaptotagmin-6 | MmAb | 55 kDa | 1 : 250 | 4 µg/ml |
| NN228-1 | pan-specific | Voltage-dependent L-type calcium | MmAb | 78 kDa | 1 : 250 | 4 µg/ml |
| NN345-1 | pan-specific | Synaptotagmin-12 | MmAb | 45 kDa | 1 : 250 | 4 µg/ml |
| CN0010-1 | pan-specific | b-Actin | RpAb | 42 kDa | 1 : 1000 | 1 µg/ml |
| NN194-1 | pan-specific | Amyloid Fibrils (OC) | RpAb | 50 +Fragments | 1:1000 | 1 µg/ml |
| NN198-1 | pan-specific | Amyloid Oligomers (A11) | RpAb | 50 +Fragments | 1:1000 | 1 µg/ml |
| CN001-1 | pan-specific | Beta-Actin | RpAb | 42 kDa | 1:1000 | 1 µg/ml |
