# Supplementary material for: L-Norvaline Reverses Cognitive Decline and Synaptic Loss in a Murine Model of Alzheimer’s Disease
Source: Neurotherapeutics. 2018 Oct 4;15(4):1036–54. doi: 10.1007/s13311-018-0669-5 (PMC6277292; doi:10.1007/s13311-018-0669-5)
Supplement: Supplementary file 14 — Selected results of the antibody array. Only proteins with a significant (p < 0.05) fold change from the control (CFC) are displayed (except for neuroligin, which exhibited 252% of change). The cut-off was set at ±19% change. (DOCX 19 kb) [file 13311_2018_669_MOESM8_ESM.docx]

| **Antibody Codes** | **Target Name with Alias** | **Full Target Protein Name** | **CFC (%)** | **Student T test p-value** |
| --- | --- | --- | --- | --- |
| NN294-1 | Neuroligin 1 (NLGN1) | Neuroligin-1 | 251.8 | 0.074 |
| NN317-1 | SCNN1B (Beta-ENaC; SCNEB) | Amiloride-sensitive sodium channel subunit beta | 219.7 | 0.018 |
| NK172-2 | Src | Src proto-oncogene-encoded protein-tyrosine kinase | 94.3 | 0.003 |
| NP049-1 | MYPT1 (MBS) | Protein phosphatase 1 regulatory subunit 12A | 93.5 | 0.009 |
| NP033-2 | PP2A B (PPP2R5A; B56) | Protein-serine phosphatase 2A - B regulatory subunit - B56 alpha isoform | 92.0 | 0.032 |
| NN177 | NPM1 (B23) | Nucleophosmin | 87.1 | 0.007 |
| NK099-4 | MEK1 (MAP2K1; MKK1) | MAPK/ERK dual-specificity kinase 1 | 86.0 | 0.015 |
| NK133-3 | PKCb (PRKCB1) | Protein-serine kinase C beta 1 | 84.6 | 0.017 |
| NN144-2 | PLCG1 | 1-phosphatidylinositol 4,5-bisphosphate phosphodiesterase gamma-1 | 84.3 | 0.030 |
| NK362-1 | Bcr | Breakpoint cluster region protein | 80.0 | 0.043 |
| NK163-4 | Ros (ROS1) | Orosomucoid 1 receptor-tyrosine kinase | 77.8 | 0.003 |
| NN132-2 | PHOCN (MOB4; mMOB1) | MOB-like protein phocein (Preimplantation protein 3) | 76.2 | 0.038 |
| NN102-NN124 | STAT1a | Signal transducer and activator of transcription 1 alpha | 73.6 | 0.014 |
| NN168 | ELK1 | ETS domain-containing protein Elk-1 | 71.7 | 0.032 |
| NN362-1 | VGLUT3 | Vesicular glutamate transporter 3 | 70.8 | 0.012 |
| NN436-1 | eNos (NOS3) | Nitric oxide synthase, endothelial | 67.9 | 0.041 |
| NN443-1 | Rad17 | Cell cycle checkpoint protein RAD17 | 67.3 | 0.003 |
| NK090-1 | Ksr1 | Protein-serine kinase suppressor of Ras 1 | 59.0 | 0.036 |
| NK178-2 | TrkA (NGFR; NTRK1) | Nerve growth factor (NGF) receptor-tyrosine kinase | 56.2 | 0.039 |
| NN101 | SPHK2 | Sphingosine kinase 2 | 56.1 | 0.036 |
| NK202 | PKG1a (PRKG1A) | cGMP-dependent protein kinase I-alpha | 54.1 | 0.003 |
| NN031 | Cyclin E1 (CCNE1) | Cyclin E1 | 53.8 | 0.015 |
| NK244-4 | Ret (c-Ret; GDNF receptor) | Proto-oncogene tyrosine-protein kinase receptor Ret | 53.2 | 0.040 |
| NK177-2 | Tlk1 | Tousled-like protein-serine kinase 1 | 52.6 | 0.029 |
| NN370-1 | Synaptophysin | Synaptophysin | 50.4 | 0.039 |
| NK022 | CaMKK (CaMKK1) | Calcium-calmodulin-dependent protein kinase kinase 1 | 49.8 | 0.026 |
| NK071 | Haspin | Protein-serine/threonine kinase haspin | 49.4 | 0.033 |
| NK201 | PKCa (PRKCA) | Protein-serine kinase C alpha | 49.4 | 0.013 |
| NN431-1 | MyoD (MYOD1) | Myoblast determination protein 1 | 47.4 | 0.035 |
| NK099-3 | MEK1 (MAP2K1; MKK1) | MAPK/ERK dual-specificity kinase 1 | 45.9 | 0.001 |
| NK111 | Mnk2 (MKNK2) | MAP kinase-interacting serine-threonine kinase 2 | 45.4 | 0.040 |
| NN105-2 | STAT5A | Signal transducer and activator of transcription 5A | 45.3 | 0.006 |
| NN300-1 | NrCAM | Neuronal cell adhesion molecule | 43.3 | 0.003 |
| NN271-1 | INPP5F (OCRL) | Inositol polyphosphate 5-phosphatase OCRL-1 | 43.0 | 0.022 |
| NK402-1 | PKAR2B | cAMP-dependent protein-serine kinase regulatory type 2 subunit beta | 42.6 | 0.038 |
| NK283-1 | MRCKb | Protein-serine/threonine kinase MRCK beta | 42.4 | 0.001 |
| NK112 | Mos | Moloney sarcoma oncogene-encoded protein-serine kinase | 42.3 | 0.012 |
| NN347-2 | TNFR1 (CD120a; TNFRSF1A; TNFAR) | Tumour necrosis factor receptor superfamily member 1A | 41.9 | 0.022 |
| NN364-3 | ACTA1 (Alpha-actin) | Actin, alpha, beta and gamma | 41.4 | 0.009 |
| NN086 | Paxillin 1 (PXN) | Paxillin 1 | 40.8 | 0.034 |
| NK400-1 | TEC | Protein-tyrosine kinase Tec | 40.3 | 0.034 |
| NN437-1 | Nrf2 (NFE2L2) | Nuclear factor erythroid 2-related factor 2 | 40.3 | 0.020 |
| NN217-1 | Beclin 1 (BECN1; GT197) | Beclin-1 | 39.8 | 0.045 |
| NN033-3 | PP2A/Bb (PPP2R2B) | Protein-serine phosphatase 2A - B regulatory subunit - beta isoform | 39.3 | 0.016 |
| NN228-1 | CavBeta2 (CACNB2; CAB2) | Voltage-dependent L-type calcium channel subunit beta-2 | 38.5 | 0.038 |
| NN390-1 | ACACA (ACC1; ACCA) | Acetyl-CoA carboxylase 1 | 37.5 | 0.032 |
| NN405-1 | DAB1 | Disabled homologue 1 | 37.4 | 0.015 |
| NN364-2 | ACTA1 (Alpha-actin) | Actin, alpha skeletal muscle | 35.2 | 0.049 |
| NK155-9 | Raf1 (c-Raf) | RAF proto-oncogene serine/threonine-protein kinase | 33.8 | 0.030 |
| NK172-3 | Src | Src proto-oncogene-encoded protein-tyrosine kinase | 33.7 | 0.006 |
| NN394-1 | BLNK | B-cell linker protein | 32.6 | 0.045 |
| NN344-1 | SYT10 | Synaptotagmin-10 | 32.6 | 0.010 |
| NN106-2 | STAT5B | Signal transducer and activator of transcription 5B | 32.0 | 0.019 |
| NN438-1 | OSR1 | Protein odd-skipped-related 1 | 31.9 | 0.034 |
| NK241-2 | Kit | Mast/stem cell growth factor receptor Kit | 30.7 | 0.029 |
| NN379-1 | COX4I1 | Cytochrome c oxidase subunit 4 isoform 1, mitochondrial | 30.5 | 0.024 |
| NK134-2 | PKCb2 (PRKCB2) | Protein-serine kinase C beta 2 | 29.8 | 0.047 |
| NK133 | PKCb (PRKCB1) | Protein-serine kinase C beta 1 | 29.4 | 0.041 |
| NK281-2 | MOK | MAPK/MAK/MRK overlapping kinase | 29.4 | 0.040 |
| NK144-1 | PKR1 (PRKR; EIF2AK2) | Double stranded RNA dependent protein-serine kinase | 29.3 | 0.039 |
| NN150-3 | RAB5A (Rab5) | Ras-related protein Rab-5A | 29.2 | 0.018 |
| NN062 | HSP105 (HSPH1, HSP110) | Heat shock 105 kDa protein | 29.0 | 0.032 |
| NK211 | CaMK1a (CaMKI) | Calcium/calmodulin-dependent protein-serine kinase 1 alpha | 28.7 | 0.003 |
| NK100-5 | MEK2 (MAP2K2; MKK2) | MAPK/ERK dual-specificity kinase 2 | 28.6 | 0.037 |
| NK053 | EphA1 | Ephrin type-A receptor 1 protein-tyrosine kinase | 28.3 | 0.041 |
| NK211-2 | CaMK1a (CaMKI) | Calcium/calmodulin-dependent protein-serine kinase 1 alpha | 28.1 | 0.043 |
| NK101-7 | MKK3 (MAP2K3; MEK3) | MAPK/ERK dual-specificity kinase 3 beta isoform | 27.6 | 0.003 |
| NK123 | PAK3 (PAKb) | p21-activated kinase 3 (beta) (Protein-serine/threonine kinase PAK3) | 26.7 | 0.046 |
| NN030-1 | Cyclin D1 (CCND1) | Cyclin D1 | 25.4 | 0.031 |
| NK086-4 | JAK3 | Janus protein-tyrosine kinase 3 | 25.1 | 0.030 |
| NP013-NP014 | PP2A/Ca (PPP2CA)/'PP2A/Cb (PPP2CB) | Protein-serine phosphatase 2A - catalytic subunit - alpha and beta isoform | 24.4 | 0.022 |
| NP050-1 | PP2B-B1/2 | Calcineurin subunit B type 2 | 24.4 | 0.005 |
| NN089 | PIK3R1 (PI3K p85) | Phosphatidylinositol 3-kinase regulatory subunit alpha | 23.7 | 0.034 |
| NN171-2 | Synapsin 1 | Synapsin 1 isoform Ia | 23.6 | 0.009 |
| NK166-2 | RSK4 (RPS6KA6) | Ribosomal S6 protein-serine kinase 4 (alpha 6) | 23.6 | 0.001 |
| NN424-1 | ITGA4 (CD49D) | Integrin alpha 4 (VLA4) | 22.9 | 0.044 |
| NN281-1 | KRAS (KRAS2; RASK2) | GTPase KRas | 22.6 | 0.002 |
| NK259-2 | AMPKa1 | 5'-AMP-activated protein kinase catalytic subunit alpha-1 | 22.2 | 0.035 |
| NK288-1 | Pim2 | Protein-serine/threonine kinase pim-2 | 21.6 | 0.003 |
| NN237-1 | DICER1 (Dicer; HERNA; Helicase MOI) | Endoribonuclease Dicer | 21.1 | 0.028 |
| NK121-4 | p38d MAPK (MAPK13) | Mitogen-activated protein-serine kinase p38 delta | 20.3 | 0.023 |
| NK226-1 | VEGFR1 (Flt1) | Vascular endothelial growth factor receptor 1 | 20.1 | 0.026 |
| NP005-2 | LAR (PTPRF) | Receptor-type tyrosine-protein phosphatase F | 19.8 | 0.002 |
| NK253-2 | WNK2 (PRKWNK2) | Protein-serine/threonine kinase WNK2 | 19.7 | 0.026 |
| NK230-2 | ATM | Ataxia telangiectasia mutated | 19.7 | 0.011 |
| NN328-1 | SOD3 | Extracellular superoxide dismutase [Cu-Zn] | 19.4 | 0.041 |
| NN134 | Striatin | Striatin | -21.9 | 0.015 |
| NN052-7 | HO1 (HO; HMOX1) | Heme oxygenase 1 | -24.4 | 0.005 |
| NN312-1 | REEP1 | Receptor expression-enhancing protein 1 | -24.8 | 0.049 |
| NK291-1 | Plk4 (SAK; STK18) | Polo-like protein-serine kinase 4 | -24.8 | 0.004 |
| NP041-3 | DUSP7 | Dual specificity protein phosphatase-7 | -25.0 | 0.003 |
| NN060-12 | HSP72 | Heat shock-related 70 kDa protein 2 | -27.4 | 0.024 |
| NN061-3 | HSP90AB1 | Heat shock 90 kDa protein beta | -34.7 | 0.045 |
| NP033 | PP2A B (PPP2R5A; B56) | Protein-serine phosphatase 2A - B regulatory subunit - B56 alpha isoform | -35.6 | 0.008 |
| NK028-4 | CDK5 | Cyclin-dependent protein-serine kinase 5 | -36.3 | 0.024 |
| NN141-1 | PDI (P4hb; PDIA1; ERBA2L; PO4DB) | Protein disulfide-isomerase | -38.4 | 0.042 |
| NK085-3 | JAK2 | Janus protein-tyrosine kinase 2 | -39.4 | 0.043 |
| NN248-1 | FIH (HIF1; HIF1AN) | Hypoxia-inducible factor 1-alpha inhibitor | -44.9 | 0.017 |
| NN350-1 | TRPC5 (TRP5) | Short transient receptor potential channel 5 | -47.0 | 0.029 |
| NP039-1 | DUSP5 | Dual specificity protein phosphatase-5 | -49.1 | 0.040 |
| NK254-1 | WNK3 (PRKWNK3) | Protein-serine/threonine kinase WNK3 | -50.6 | 0.045 |
| NK129-5 | Akt1 (PKBa) | RAC-alpha protein-serine/threonine kinase | -53.4 | 0.038 |
| NN047 | Grp75 (HspA9) | Stress-70 protein, mitochondrial | -59.3 | 0.008 |
